# Ewing Sarcoma Single-cell Transcriptome Analysis Reveals Functionally Impaired Antigen-presenting Cells

**DOI:** 10.1158/2767-9764.CRC-23-0027

**Published:** 2023-10-24

**Authors:** Lindy L. Visser, Margit Bleijs, Thanasis Margaritis, Marc van de Wetering, Frank C. P. Holstege, Hans Clevers

**Affiliations:** 1 Princess Máxima Center for Pediatric Oncology, Utrecht, the Netherlands.; 2 Center for Molecular Medicine, UMC Utrecht and Utrecht University, Utrecht, the Netherlands.; 3 Hubrecht Institute, Royal Netherlands Academy of Arts and Sciences (KNAW) and University Medical Centre, Utrecht, the Netherlands.

## Abstract

Novel therapeutic strategies are urgently needed for patients with high-risk Ewing sarcoma and for the reduction of severe side effects for all patients. Immunotherapy may fill this need, but its successful application has been hampered by a lack of knowledge on the composition and function of the Ewing sarcoma immune microenvironment. Here, we explore the immune microenvironment of Ewing sarcoma, by single-cell RNA sequencing of 18 Ewing sarcoma primary tissue samples. Ewing sarcoma is infiltrated by natural killer, T, and B cells, dendritic cells, and immunosuppressive macrophages. Ewing sarcoma–associated T cells show various degrees of dysfunction. The antigen-presenting cells found in Ewing sarcoma lack costimulatory gene expression, implying functional impairment. Interaction analysis reveals a clear role for Ewing sarcoma tumor cells in turning the Ewing sarcoma immune microenvironment into an immunosuppressive niche. These results provide novel insights into the functional state of immune cells in the Ewing sarcoma tumor microenvironment and suggest mechanisms by which Ewing sarcoma tumor cells interact with, and shape, the immune microenvironment.

**Significance:**

This study is the first presenting a detailed analysis of the Ewing sarcoma microenvironment using single-cell RNA sequencing. We provide novel insight into the functional state of immune cells and suggests mechanisms by which Ewing tumor cells interact with, and shape, their immune microenvironment. These insights provide help in understanding the failures and successes of immunotherapy in Ewing sarcoma and may guide novel targeted (immuno) therapeutic approaches.

## Introduction

Ewing sarcoma (EwS) is a highly aggressive solid tumor and the second most common malignant bone tumor in adolescents and young adults, often occurring during the growth spurt (1). This sarcoma arises in bone and soft tissue and is most frequently located in the pelvis, femur, tibia, ribs, thoracic wall, gluteal muscle, pleural cavities, and cervical muscles (2). Genetically, Ewing sarcoma is characterized by a (single) genomic translocation, which results in the expression of a gene fusion involving *EWSR1* and an *ETS* family transcription factor, most frequently *FLI1* (1). The chimeric *EWS-FLI1* transcription factor affects target gene expression in several ways, including through alteration of DNA methylation, histone modifications, and noncoding RNA expression (3).

In recent years, significant strides have been made in treating children with Ewing sarcoma. Standard treatment, consisting of chemotherapy, surgery, and local radiotherapy, is reaching a 5-year overall survival (OS) rate of approximately 75% (4). Yet, prognosis of patients with metastatic or relapsed disease remains dismal (<30%) and has not improved in decades (4). On top of that, current treatment is detrimental for health and can result in long-term disability, thus reducing the pediatric patient's quality of life. Therefore, there is an urgent need for new therapeutic strategies.

Immunotherapy has shown promise for treating pediatric cancers, as highlighted by its success in leukemia, lymphoma, and to some extent in neuroblastoma (5–8). Clinical success in solid tumors has been limited (9). Similar to other pediatric tumors, Ewing sarcoma is considered to have low immunogenicity, because of its low mutational burden (10). Pediatric sarcomas have also been suggested to activate immunosuppressive mechanisms within the tumor immune microenvironment, further reducing immunotherapeutic efficacy (11). Immunotherapeutic approaches explored for Ewing sarcoma (including checkpoint inhibition, mAbs, cancer vaccines, and genetically engineered T cells) have shown varying preclinical successes and few have made it into clinical testing (12, 13).

Immunotherapy studies are hampered by a lack of knowledge on the composition and function of the Ewing sarcoma immune microenvironment. Previous IHC and bulk transcriptome-based studies have shown that the Ewing sarcoma immune microenvironment is dominated by immature, or undifferentiated, macrophages and immunosuppressive M2-like macrophages (14, 15). In addition, a scarcity of infiltrated cytotoxic T cells, mature dendritic cells (DC), and proinflammatory M1-like macrophages have been found, but a detailed molecular and cellular characterization of the Ewing sarcoma tumor microenvironment (TME) is lacking.

To gain insight into the composition and function of the Ewing sarcoma immune microenvironment, we profiled 18 Ewing sarcoma samples from 11 different patients, taken before and after treatment, using single-cell RNA sequencing (scRNA-seq). We provide a comprehensive overview of the Ewing sarcoma immune microenvironment and identify several immune cell subsets with immunosuppressive and functionally impaired phenotypes. Using cell–cell interaction analysis, we find a clear role for Ewing sarcoma tumor cells in shaping the Ewing sarcoma immune microenvironment into an immunosuppressive niche, thereby providing new rationales for (immuno)therapeutic approaches.

## Materials and Methods

### Patient-derived Ewing Sarcoma Specimen

Surgically resected tissue was obtained from patients with Ewing sarcoma at the Princess Máxima Center, via an established sample acquisition route as part of a biobank initiative (remaining tumor tissue). Ethical approval was granted for the biobanking initiative by the Medical Research Ethics Committee of the University Medical Center Utrecht, and the Máxima biobank committee granted approval for the present study. All patients and/or their legal representatives signed informed consent for their tumor samples being used for research purposes. Experiments conformed to the principles stated in the Declaration of Helsinki and the Department of Health and Human Services Belmont Report.

Eighteen primary tissue samples were collected from 11 patients (including 2 females and 9 males; 6–17 years old), including both treatment-naïve and treatment-exposed samples (Supplementary Table S1). All samples had a detectable *EWS-FLI1* gene fusion. Tumor material was washed with basal medium containing Advanced DMEM F12 (AdDMEM-F12; Thermo Fisher Scientific, #12634010) supplemented with 1% penicillin/streptomycin (Thermo Fisher Scientific, #15140122), 1% Glutamax (Thermo Fisher Scientific, #35050038), and 1% Hepes (Thermo Fisher Scientific, #15630056) and minced into tumor pieces. The tumor pieces were washed with AdDMEM-F12 and a part of the tumor pieces was frozen in AdDMEM-F12 supplemented with 50% FCS (Gibco, #16140071) and 10% DMSO (Sigma-Aldrich, #D8418).

### Tissue Dissociation and scRNA-seq

The primary Ewing sarcoma material was minced using scalpels and mechanically disrupted by pipetting up and down. Single cells were taken off and kept apart on ice. Left over tissue clumps were digested at 37°C using digestion buffer, containing basal medium supplemented with 1 mg/mL Collagenase1A (Sigma-Aldrich, #C9891) and 100 U/mL DNAse I (Roche, #4716728001) for three 10 minutes rounds; after each round, single cells were collected and kept on ice and fresh digestion buffer was added to the leftover clumps. After digestion, red blood cell lysis buffer (Sigma-Aldrich, #11814389001) was applied for 5 minutes to lyse the red blood cells. The single cells were washed with FACS buffer, containing PBS supplemented with 2% FCS, 2 mmol/L ethylenediamine tetraacetic acid (EDTA), and 100 U/mL DNAse I. The cells were stained for 30 minutes with Human CD45 PE-conjugated Antibody (R&D Systems, #FAB1430P-100) in FACS buffer.

Prior to sorting, single-cell suspensions were stained in 5 µmol/L DRAQ5 and 1 µmol/L DAPI (Sigma-Aldrich, #D9542). Viable single cells were sorted on the basis of forward/side-scatter properties, DAPI-, DRAQ5-, and CD45 staining, as shown in Supplementary Fig. S1, using FACS (SONY SH800S Cell Sorter) into 384-well plates (Bio-Rad) containing 10 µL of mineral oil (Sigma) and 50 nL of RT primers. Libraries were prepared using the CEL-Seq2 method (16, 17) and subjected to paired-end sequencing with 75-bp length using the Illumina NextSeq500 sequencer.

### Mapping, Filtering, and scRNA-seq Analysis

The Sharq pipeline was used to process the sequencing data as described previously (18). Mapping was performed using STAR (version 2.6.1), on the Genome Reference Consortium GRCh38. Read assignment was performed with featureCounts (version 1.5.2), using a gene annotation based on GENCODE version 26. Unique transcript counts were normalized using sctransform and analyzed using the Seurat R package (version 4.0.1). More details on the data cleanup and analysis, including cluster annotation and used gene sets/signatures, can be found in the Supplementary Materials and Methods.

### Statistical Analysis

Comparisons between two groups were performed using the Wilcoxon rank-sum test, where comparison of gene expression additionally included the Bonferroni correction. Comparison of treatment-naïve and treatment-exposed samples was performed using the Mann–Whitney *U* test. Statistical comparisons between three or more groups were performed using the Kruskal–Wallis test with Dunn *post hoc* test for multiple comparisons. All statistical analyses were performed using R (version 4.0.1). *P* values ≤0.05 were considered statistically significant.

### Data Availability

The scRNA-seq data of the Ewing sarcoma primary samples, generated for the purpose of this study, is made available in the Gene Expression Omnibus (GEO). The GEO accession number belonging to this data is GSE243347 (“Ewing sarcoma single-cell transcriptome analysis reveals functionally impaired antigen-presenting cells”). The neuroblastoma scRNA-seq data used for comparison in this study were obtained from https://www.neuroblastomacellatlas.org/, which contains the Neuroblastoma Princess Màxima Center dataset as presented by Kildisiute and colleagues (19).

## Results

### Single-cell Architecture of Primary Ewing Sarcoma

**FIGURE 1:**
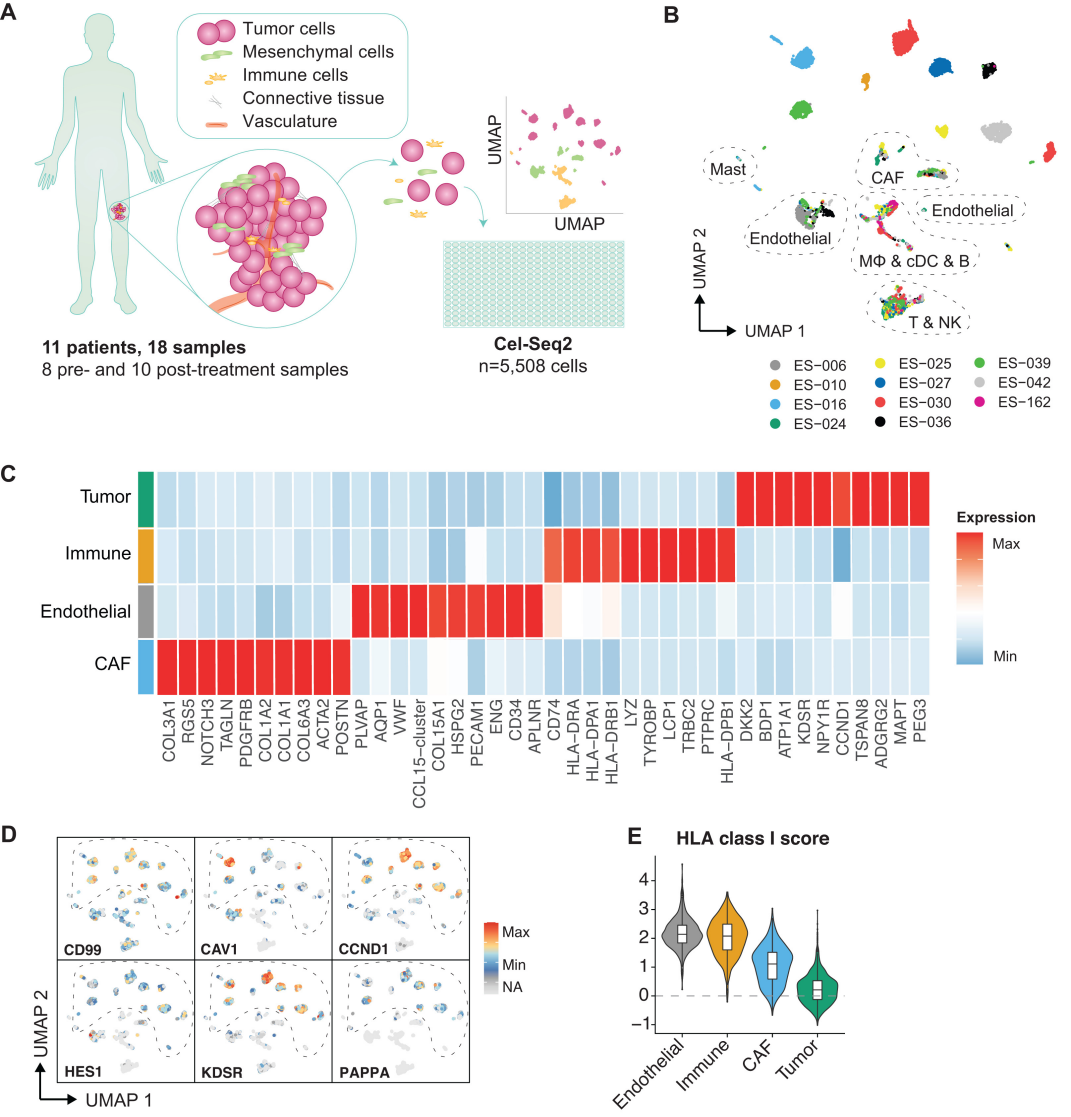
Single-cell architecture of primary Ewing sarcoma. **A,** Graphical overview of primary patient-derived tissue dissociated and sorted for scRNA-seq. **B,** Uniform Manifold Approximation and Projection (UMAP) plot colored by patient origin. **C,** Heat map of top 10 differentially expressed genes per main cell type by average log-normalized expression. **D,** UMAP with expression of Ewing diagnostic marker (*CD99*) and a selection of *EWS-FLI1* candidate target genes (*CAV1, CCND1, HES1, KDSR, PAPPA*). Dotted line highlights the tumor cell clusters. NA: not expressed. **E,** Feature plots showing HLA class I module scores (*HLA-A, HLA-B, HLA-C, HLA-E, HLA-F, B2M*) of main cell types.

To obtain a detailed overview of the cellular composition of Ewing sarcoma, 18 primary tumor samples were analyzed using scRNA-seq. Because of the limited amount of material (i.e., small needle biopsies) in some cases, a plate-based scRNA-seq method, CEL-Seq2 (16, 17), was applied, whereby both tumor and immune cells were well represented. scRNA-seq yielded 5,508 high-quality cells (Table 1; Fig. 1A). One sample was excluded at an early stage (ES-048), because of a low cell number (10 cells).

**TABLE 1:**
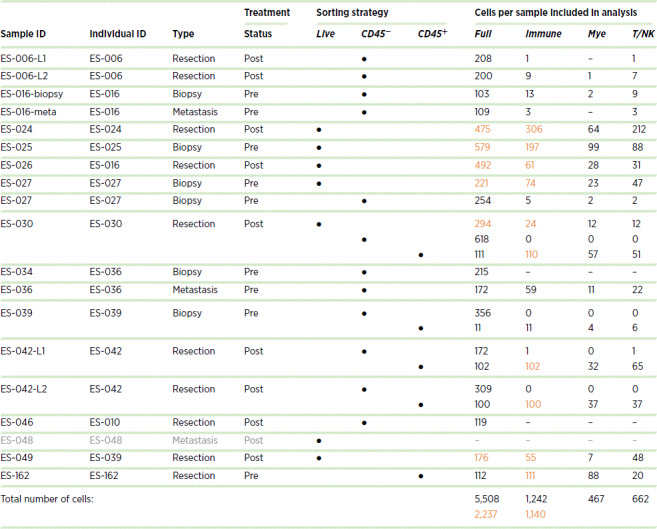
Overview of cells per sample included in analysis.

**NOTE:** Orange font: included in composition analysis. Gray font: Sample excluded at early stage, because of low number of high-quality cells.

Unbiased clustering analysis using the Louvain algorithm identified 20 distinct cell clusters, which were annotated as four main cell types, that is, tumor cells, endothelial cells, cancer-associated fibroblasts (CAF), and immune cells [natural killer (NK), T and B cells, macrophages (Mφ), DCs, and mast cells; Fig. 1B; Supplementary Fig. S2A–S2C]. The cellular composition of the tumors varied between the different samples, but no distinct changes were observed when comparing treatment-naïve and treatment-exposed samples (Supplementary Fig. S2D and S2E).

The CAF clusters represented two distinct subsets: cancer-associated myofibroblasts (*ACTA2, MEF2C, TBX2*) and inflammatory/adipogenic CAFs (*FAP, CFD, CREB2L1*), following the classification proposed by Luo and colleagues (ref. 20; Supplementary Fig S3A–S3E). The CAFs were mainly originating from three samples, with variable sample locations, and the CAF subsets were not associated with treatment status (Supplementary Fig. S2E, S3B, and S3C; Supplementary Table S1).

The tumor clusters expressed several genes that are known to be expressed by Ewing sarcoma tumor cells, including the diagnostic marker *CD99* and *EWS-FLI1* candidate target genes *CAV1*, *CCND1*, *HES1*, *KDSR*, and *PAPPA* (Fig. 1C and D; Supplementary Fig. S2F and S4A; refs. 21–23). Tumor cell identity was further confirmed by copy-number variation inference, which showed typical chromosomal aberrations (e.g., gains in chromosomes 1q, 2q, and 8; Supplementary Fig. S4B; ref. 2). Interestingly, the Ewing sarcoma tumor cells of some individual patients clustered into several subpopulations, for example, patient ES-030 (treatment-exposed sample) in clusters 1 and 10, patient ES-025 (treatment-naïve sample) in clusters 13 and 21 and patient ES-039 (treatment-naïve sample) in clusters 7 and 26, indicative of intratumor heterogeneity (Fig. 1B; Supplementary Fig. S2A and S2B). Finally, in line with previous findings in cell lines and primary samples (24), HLA class I expression was significantly lower in malignant than in nonmalignant cells, highlighting the low immunogenicity of Ewing sarcoma (Fig. 1E; Supplementary Fig. S2G–S2H).

### Ewing Sarcoma–associated Lymphocytes Display Variable Degrees of Dysfunctionality and Immune Suppression

Tumor-infiltrating immune cells are important components of the TME as they can both promote or inhibit cancer progression. To characterize the composition and functional state of Ewing sarcoma–infiltrating immune cells, we performed an in-depth analysis of the immune clusters. Among the 1,242 immune cells, we identified myeloid and lymphoid cells (Table 1; Fig. 1A; Supplementary Fig. S5A and S5B). The composition of immune cell populations varied among the different Ewing sarcoma samples, but no distinct changes were observed when comparing treatment-naïve and treatment-exposed samples (Supplementary Fig. S5C). When comparing treatment-naïve and treatment-exposed Ewing sarcoma samples, we found a relative increase in T lymphocyte populations and a corresponding decrease in macrophages in treatment-exposed samples (*P* = 0.1; Supplementary Fig. S5D).

**FIGURE 2:**
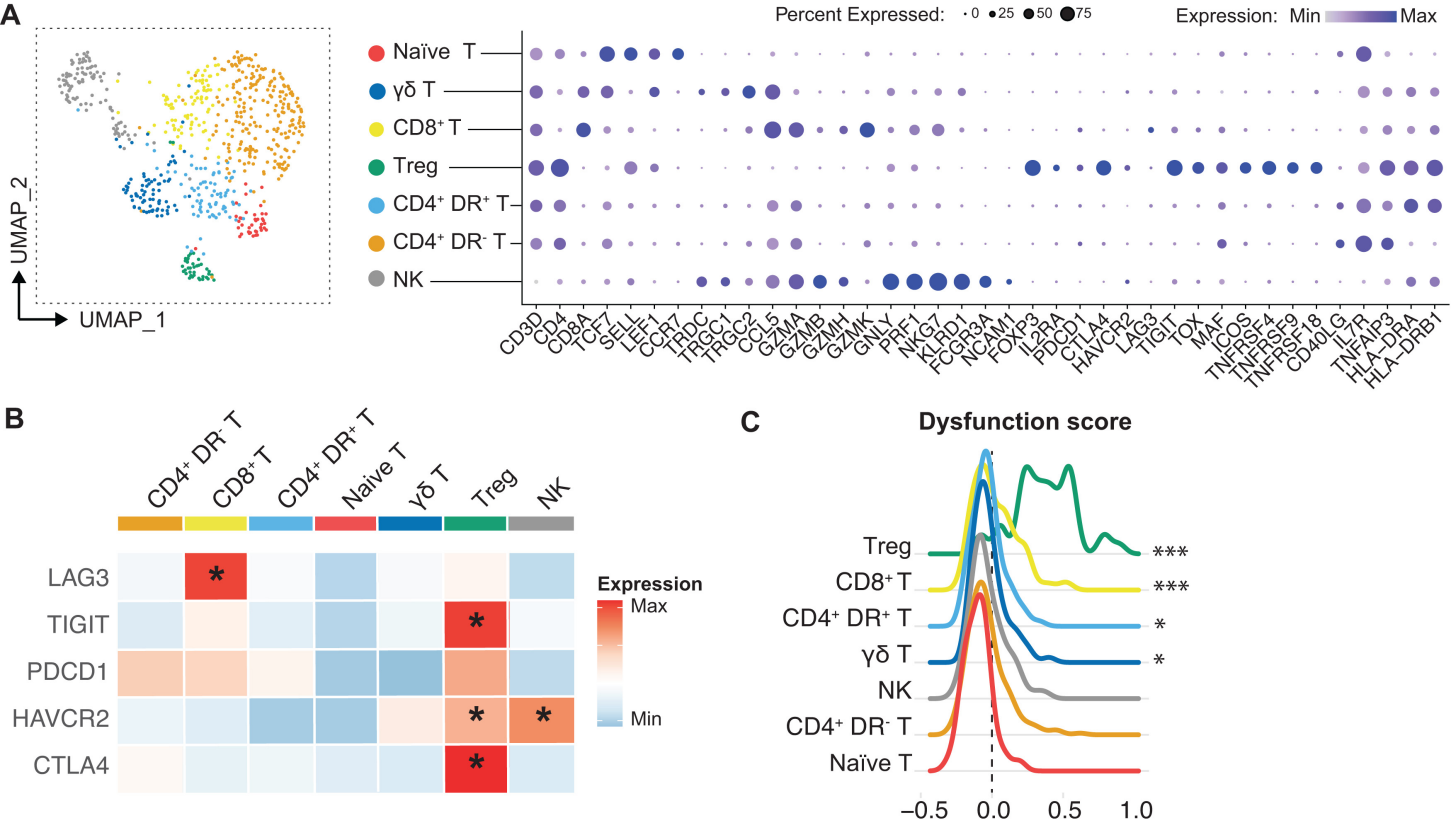
Ewing sarcoma–associated lymphocytes display variable degrees of dysfunctionality and immune suppression. **A,** UMAP and dotplot of the T- and NK-cell subsets and a selection of their marker genes. **B,** Heat map showing average expression of inhibitory receptors in the T- and NK-cell subsets. *Significantly upregulated with *P* < 0.0001 using Wilcoxon rank-sum test with Bonferroni correction. **C,** Dysfunction scores constructed using genes from B. Cell types ordered from high to low score. *, *P* < 0.05; ***, *P* < 0.001 versus Naïve T.

The lymphocyte compartment consisted of NK cells, various T-cell subsets, and a small fraction of B cells (Table 1; Fig. 1B and 2A; Supplementary Fig. S6A and S6B). Ewing sarcoma NK cells were mostly of the CD56^dim^ CD16^+^ type type (genes *NCAM1* and *FCGR3A*) and expressed genes encoding transcription factors associated with terminal differentiation and effector function [TBX21 (T-bet) and ZEB2; Fig 2A] (25). The following T-cell subsets were identified in the Ewing sarcoma TME: cytotoxic T cells (CD8^+^ T), γδ T cells, naïve T cells, Th cells (CD4^+^ T), and regulatory T cells (Treg). CD4^+^ T cells were the most abundant and consisted of two subpopulations: one expressing high HLA-DR (CD4^+^ DR^+^ T), a late T-cell activation marker, and a second with low expression of HLA-DR (CD4^+^ DR^−^ T). The HLA-DR^+^ CD4^+^ T-cell subset was mainly found in treatment-exposed samples (Supplementary Fig. S5C), suggesting that a treatment-induced immune response had taken place.

To assess whether the T cells found in our Ewing sarcoma samples are actual tumor-associated T cells, previously reported T-cell gene signatures specific for either tumor- or blood/normal tissue-associated T cells were utilized. Gene set enrichment analysis (GSEA) was performed to assess the resemblance, expressed in normalized enrichment score (NES), between the differentially expressed genes of our Ewing sarcoma T-cell subsets and the published T-cell signatures (Supplementary Materials and Methods). This indicated that CD8^+^ T and Tregs showed high resemblance (high NES score) with tumor-associated T-cell signatures (CD8^+^ GZMK^+^ effector memory and CD4^+^ Tregs, respectively), whereas the CD4^+^ T populations resembled tumor/normal Th17 cells and naïve T-cell signatures (Supplementary Fig. S6C; ref. 26). In line with this, Tregs expressed high levels of their marker genes *FOXP3* and *IL2RA*, in addition to the transcription factor *MAF* and checkpoint genes *ICOS*, *TNFRSF4* (OX‐40), *TNFRSF9* (4-1BB), and *TNFRSF18* (GITR; Fig. 2A). Expression of these transcription factor and checkpoint genes is upregulated in tumor-associated/infiltrated Tregs and has been linked with enhanced inhibitory capacity (27). CD8^+^ T cells and NK cells were characterized by a high expression of cytotoxic genes (CD8: *GZMA*, *GZMH*, *GZMK, CCL5*; NK: *GNLY, PRF1, GZMB*), indicating their cytotoxic properties (Fig. 2A; Supplementary Fig S6D). However, a subset of these cells also showed significant increased expression of dysfunction markers [CD8: *LAG3;* NK: *HAVCR2* (TIM-3); Fig. 2A and B; Supplementary Fig S6E] and had a high dysfunction score (Fig. 2C), indicating the presence of a dysfunctional subpopulation among these lymphocyte subsets. These results show that lymphocytes in Ewing sarcoma display variable degrees of dysfunctional and immune suppression.

### Ewing Sarcoma is Infiltrated by Immunosuppressive Macrophages and Functionally Impaired Antigen-presenting Cells

**FIGURE 3:**
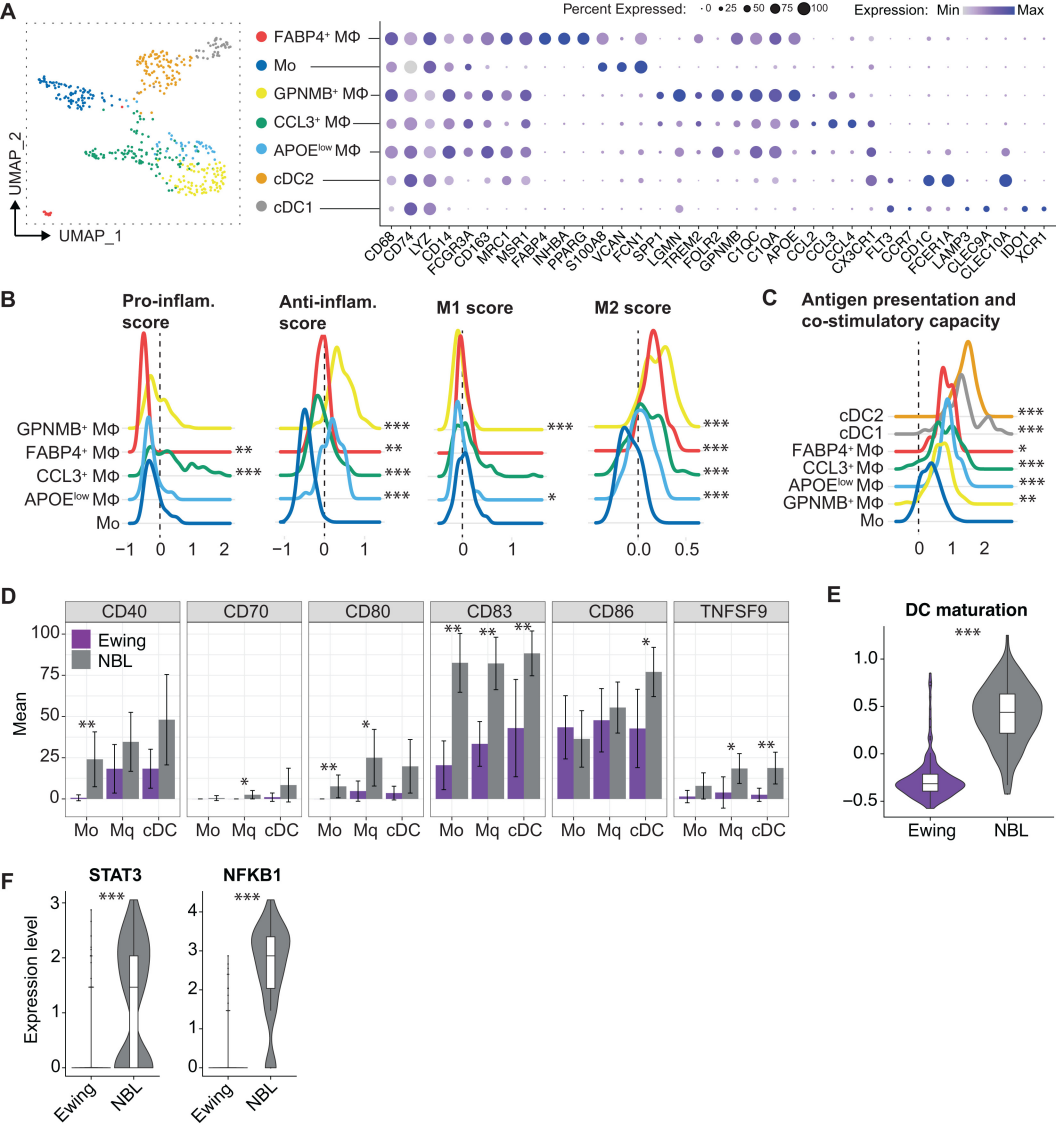
Ewing sarcoma is infiltrated by immunosuppressive macrophages and functionally impaired APCs. **A,** UMAP and dotplot of the myeloid compartment, showing conventional dendritic cells type 1 and 2 (cDC1, cDC2), undifferentiated macrophages (Mo), and differentiated macrophage populations (Mφ) and a selection of their marker genes. **B,** Macrophage-specific scores for proinflammatory and anti-inflammatory signatures as well as M1 and M2 signatures. **C,** Antigen presentation and costimulatory capacity score as constructed with genes shown in Supplementary Fig. S7D. *, *P* < 0.05; **, *P* < 0.01; ***, *P* < 0.001 versus Mo. **D,** Barplot showing percentage of costimulatory gene-expressing Mo, Mφ, and cDCs. Neuroblastoma (NBL; Kildisiute and colleagues) data included as comparative group. Mean percentages and error bars are plotted for individual samples. Samples with <5 cells per group were excluded. *, *P <* 0.05; **, *P* < 0.01; ***, *P* < 0.001. **E,** Violin plot depicting DC maturation score for Ewing sarcoma and NBL cDCs. Z-scores were calculated using a curated MSigDB gene set (“LINDSTEDT_DENDRITIC_CELL_MATURATION_B”). This gene set includes upregulated genes both at 8 and 48 hours in response to inflammatory stimuli. ***, *P* < 0.001. **F,** Violin plots showing the *STAT3* and *NFKB1* gene expression levels of Ewing sarcoma and NBL cDCs. ***, *P* < 0.001 with Bonferroni correction.

Myeloid cells are key regulators of antitumor immunity because of their involvement in antigen presentation and T-cell activation, but can also induce immune suppression (28). The myeloid compartment of Ewing sarcoma was composed of mast cells, a small fraction of plasmacytoid dendritic cells (pDC), type 1 and type 2 conventional dendritic cells (cDC1, cDC2), undifferentiated macrophages (Mo), and four differentiated macrophage subsets (FAPB4^+^, CCL3^+^, APOE^low^, and GPNMB^+^ Mφ; Table 1; Fig. 2A and 3A). The FABP4^+^ Mφ subset has been previously identified as an alveolar macrophage population in the lungs (29). Indeed, we found this population of macrophages in patient sample ES-042 (Supplementary Fig. S7A), resected from the thoracic area, further verifying the sensitivity of our approach.

Macrophages can display a variety of activation states, including proinflammatory M1-like and immunosuppressive M2-like macrophages (30). Compared with Mo, all Mφ subsets showed a high M2-like and high anti-inflammatory signature (Fig. 3B), associated with an immunosuppressive and protumorigenic phenotype. These immunosuppressive macrophage populations were well represented in all patient samples (Supplementary Fig. S7A). CCL3^+^ Mφ also showed a high proinflammatory signature compared with Mo (Fig. 3B; Supplementary Fig. S7C and S7D). The CCL3^+^ Mφ subset was composed of cells derived from patient ES-025 and ES-026 (Supplementary Fig. S7A), indicating that these patients had an enrichment of the proinflammatory macrophage population, as in most Ewing sarcoma patient samples they were of low abundance.

To assess the antigen-presenting and costimulatory capacity of the antigen-presenting cells (APC; macrophages and cDCs), a gene signature was used including HLA class I and II genes and genes important for the costimulation of T cells (Fig. 3C; Supplementary Fig. S7D). cDCs had the highest signature score, making them the most potent T-cell interactors, followed by the Mφ subsets. Surprisingly, compared with another pediatric cancer type, neuroblastoma (19), Ewing sarcoma contained only a small percentage of costimulatory gene-expressing APCs (Fig. 3D; Supplementary Fig. S7D). Ewing sarcoma cDCs showed significantly lower percentages of *CD83*-, *CD86*-, and *TNFSF9-*expressing cells and Ewing sarcoma Mφ had significantly lower *CD70*-, *CD80-*, *CD83*-, and *TNFSF9*-expressing cells, than neuroblastoma. This suggests that Ewing sarcoma APCs—and especially Ewing sarcoma cDCs—are unable to effectively stimulate T cells, and thus are functionally impaired, resulting in T-cell anergy (31). Downsampling of the data to account for differences in sequencing depth did not alter the results (Supplementary Fig. S8A). Using a curated gene set for DC maturation (Supplementary Materials and Methods), Ewing sarcoma cDCs furthermore showed a significantly lower maturation score as compared with neuroblastoma cDCs, supporting a premature phenotype (Fig. 3E). cDCs dysfunction has been previously described in non–small cell lung cancer, and attenuated NFκB and STAT3 signaling has been suggested to be the leading cause (32). NFκB and STAT3 are critical for the regulation of HLA, costimulatory molecules, and cytokines and for the regulation of immune tolerance (33, 34). Indeed, compared with neuroblastoma, in Ewing sarcoma cDCs showed significantly lower expression of *NFKB1* and *STAT3*, confirming their dysfunctional state and involving previously reported signaling pathways in the observed phenotype (Fig. 3F). In line with this, GSEA showed lower activity of inflammatory signaling (inflammatory response, IL-, IFN-, TNFα-, and IL6-JAK-STAT3 signaling) in Ewing sarcoma cDCs than in neuroblastoma cDCs, but higher oxidative phosphorylation. The latter is associated with immature/tolerogenic cDCs (35), which further supports our hypothesis of dysfunctional cDCs in Ewing sarcoma (Supplementary Fig. S8B).

In addition to the low percentage of costimulatory gene-expressing Mφ in Ewing sarcoma, these cells showed high IFN response signatures compared with neuroblastoma Mφ (Supplementary Fig. S8C). These signatures included key drivers of the type I/III IFN antiviral response, such as *IFI6*, *IFITM2,* and *MX1* (Supplementary Fig. S8D). The high IFN response signatures could be the result of sustained proinflammatory cytokine release in the Ewing sarcoma TME, which is known to eventually lead to immune suppression and tolerance (36). Taken together, these results indicate that Ewing sarcoma is infiltrated by immunosuppressive macrophages and functionally impaired APCs.

### Immunoregulatory Interactions Shape the Immunosuppressive Microenvironment of Ewing Sarcoma

**FIGURE 4:**
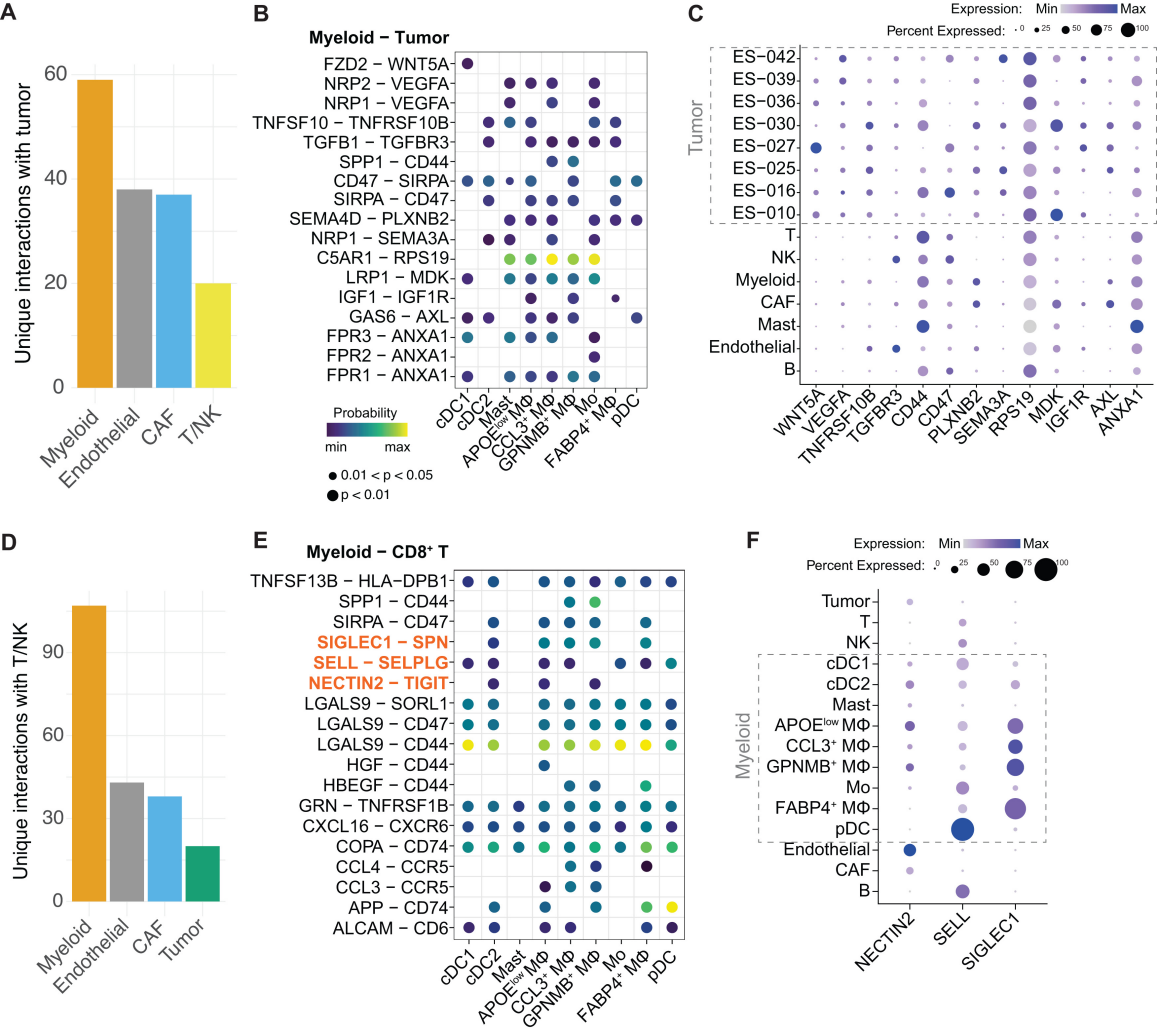
Immunoregulatory interactions shape the immunosuppressive microenvironment of Ewing sarcoma. **A,** Number of unique interactions between tumor cells and indicated main cell types. **B,** Bubble plot of predicted immunosuppressive interactions between indicated myeloid subsets and tumor cells. For full overview see Supplementary Fig. S9A. **C,** Dotplot of genes included in the selected, predicted interactions, shown in B, and expressed by the main cell types and per-patient tumor cells. **D,** Number of unique interactions between T/NK cells and indicated main cell types. **E,** Bubble plot of predicted interactions between indicated myeloid subsets and CD8^+^ T cells and are correlating (>0.5) with CD8^+^ T-cell dysfunction score. **F,** Dotplot of genes included in T cell dysfunction–promoting interactions, highlighted in orange in E, and expressed by the myeloid cells.

The immunosuppressive state of the myeloid compartment can result from suppressive cues involving molecules expressed or secreted by the Ewing sarcoma tumor cells. To unravel these cues, ligand–receptor interactions between Ewing sarcoma tumor cells and the microenvironment were modeled using CellChat (Supplementary Materials and Methods). This indicated that Ewing sarcoma tumor cells display a multitude of interactions with other cell types (Fig. 4A). Predicted interactions between myeloid cells and Ewing sarcoma tumor cells included interactions involved in cellular adhesion, chemoattraction, and immune regulation (Supplementary Fig. S9A). Several of these interactions have been ascribed an immunosuppressive role (Fig. 4B; Supplementary Fig. S9B): *FZD2*—*WNT5A* and *TGFB1*—*TGFBR3* can modulate the function of cDCs by dampening their APC functions and skewing them to an unconventional phenotype with tolerogenic features (Supplementary Table S2). *NRP1/2*—*VEGFA*, *SPP1*—*CD44*, *SEMA4D*—*PLXNB2* (CD100—CD72), *NRP1*—*SEMA3A*, *LRP1*—*MDK*, *IGF1*—*IGF1R*, and *FPR1/2/3*—*ANXA1*, among others, have been described to trigger the polarization of macrophages toward the M2 immunosuppressive phenotype, and regulate tumor-associated macrophage infiltration into hypoxic tumor regions (Supplementary Table S2). Finally, *TNFSF10*—*TNFRSF10B* (TRIAL—TRIAL-R), *SIRPA*—*CD47* (“don't eat me”), *C5AR1*—*RPS19*, and *GAS6*—*AXL* act as negative regulators of the innate immune system by inhibiting cytokine responses, phagocytosis of cancer cells, and promoting the immunosuppressive TME (Supplementary Table S2). The secreted protein genes *WNT5A*, *VEGFA,* and *SEMA3A*, and the receptor genes *AXL*, *TNFRSF10B* and *IGF1R* were mainly expressed by tumor cells (Fig. 4C). Tumor-expressed ligands and receptors were expressed at various levels by the tumor cell clusters and varied between patients.

Assessment of the interactions between tumor cells and T/NK cells did not point to a single clear cause of T/NK cell dysfunction (Fig. 4A; Supplementary Fig. S9C). Therefore, the 97 unique interactions between T/NK cells and the immunosuppressive myeloid cells were further assessed (Fig. 4D; Supplementary Fig. S9D; Supplementary Table S3). An unbiased approach was used to reduce this list of interactions to those associated with CD8^+^ T-cell dysfunction. This resulted in the identification of 18 interactions that were positively correlating with the dysfunction scores of CD8^+^ T cells (Fig. 4E; Supplementary Table S4). Among these, several interactions have been described to promote T-cell exhaustion/dysfunction: *NECTIN2*—*TIGIT*, *SELL*—*SELPLG*, and *SIGLEC1*—*SPN* (Supplementary Table S2). Assessment of *NECTIN2* expression across all cell types, showed that this gene was mainly expressed by endothelial cells, cDCs, the immunosuppressive Mφ populations (APOE^low^ Mφ and GPNMB^+^ Mφ; Fig. 4F; Supplementary Fig. S9E). *SIGLEC1* was mainly expressed by all Mφ populations, and *SELL* was expressed mainly by pDC.

**FIGURE 5:**
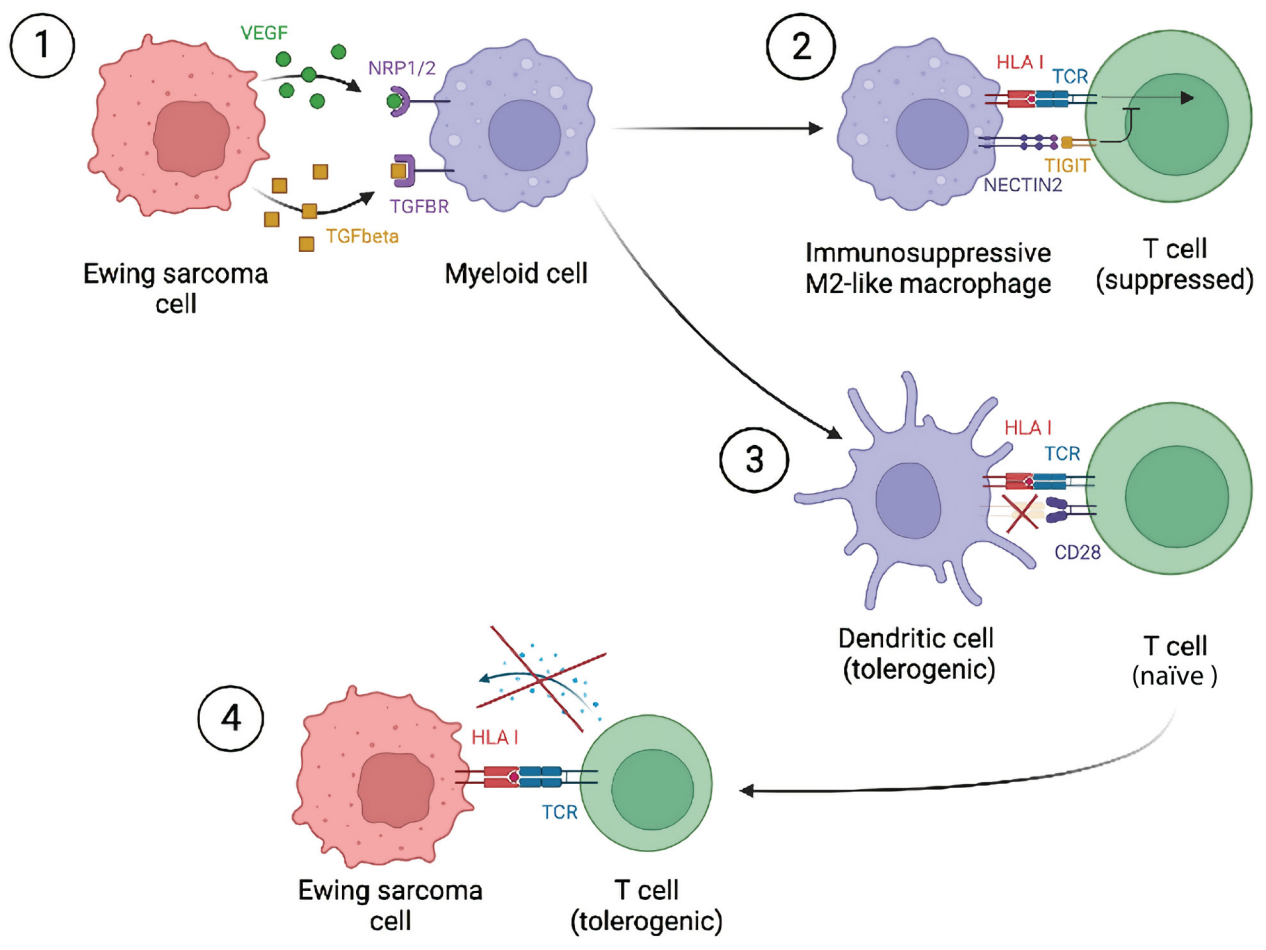
Schematic overview mechanism of Ewing sarcoma cells shaping the immune microenvironment into an immunosuppressive niche. (1) Immunomodulatory interactions between Ewing sarcoma cells and myeloid cells induce polarization of macrophages to an immunosuppressive, M2-like, phenotype and induce tolerogenic DCs. (2) Immunosuppressive macrophage interacts with T cell, where, for example, TIGIT–NECTIN2 interactions leads to the inhibition of T-cell activation resulting in a suppressed/dysfunctional T cell. (3) Tolerogenic DC interacts with naïve T cells but is lacking expression of costimulatory molecules, important for T-cell priming, resulting in a tolerogenic T cell. (4) T cell interacts with Ewing sarcoma cell, but because of it tolerogenic state the T cell is unable to activate, produce cytokines, and proliferate which are essential for cancer cell killing. Image created using BioRender.

These results implicate a clear role for Ewing sarcoma tumor cells in shaping the Ewing sarcoma immune microenvironment into an immunosuppressive niche. The predicted tumor–myeloid interactions support the immunosuppressive state of macrophages/cDCs and indicate a role for Ewing sarcoma tumor cells in directly shaping the myeloid compartment. In addition, the results imply an indirect role for Ewing sarcoma tumor cells in suppressing T-cell effector function via interactions between T/NK cells and immunosuppressive myeloid cells (Fig. 5) and provide new therapeutic opportunities, as discussed in the next section.

## Discussion

This study comprises single-cell transcriptomic profiling of 18 Ewing sarcoma primary tissue samples. Despite previous efforts to characterize Ewing sarcoma at single-cell level in cell lines or in four patient samples (22, 37, 38), the composition and function of the Ewing sarcoma immune microenvironment have remained largely unexplored. This study is the first presenting a detailed analysis of the Ewing sarcoma immune microenvironment using scRNA-seq. Our study reveals that Ewing sarcoma is infiltrated by immunosuppressive myeloid cells and Ewing sarcoma–associated T cells with various degrees of dysfunction. Remarkably, the APCs present in Ewing sarcoma show characteristics of functional impairment. Cell–cell interaction analysis reveals a clear role for Ewing sarcoma tumor cells in shaping the Ewing sarcoma immune microenvironment into an immunosuppressive niche. These findings are highly relevant for immunotherapy development for Ewing sarcoma, as they provide insight into the functional state of immune cells in the Ewing sarcoma TME, and suggest mechanisms by which Ewing sarcoma tumor cells interact with, and shape, the immune microenvironment. Together, these findings may guide novel targeted (immuno)therapeutic approaches.

Our analyses reveal that Ewing sarcoma cDCs largely lack expression of important T-cell costimulatory genes. cDCs are crucial players in the activation of antigen-specific T cells, a pivotal step in the initiation of the innate and adaptive immune response, which is essential for tumor cell clearance. However, when costimulatory molecules are absent from the cDC surface, these cells will induce an anergy-promoting environment (31, 39). This functional impairment of cDCs has been observed in several cancer types, where it ranges from influences of the tumor on the differentiation of cDCs from hematopoietic precursors, to the effect on the behavior of fully differentiated cDCs. Yet, this is the first time that cDC dysfunction has been described in pediatric cancer, and more specifically, in Ewing sarcoma.

Tumor-induced cDC dysfunction plays an important role in cancer immune escape, but the underlying mechanism is not fully understood. Proper differentiation and maturation of DCs can be disrupted by inflammatory signals. To date, several growth factors and proinflammatory cytokines involved in the abnormal differentiation of tumor-induced DCs have been described previously (40). Our analyses show that Ewing sarcoma tumor cells express *VEGFA* (Fig. 4C). Previous studies have shown that Ewing sarcoma tumor cells indeed secrete VEGF, and that elevated levels can even be detected in serum of patients with Ewing sarcoma (41, 42). *In vivo* administration of VEGF in tumor-free mice has been shown to lead to impaired cDC development (43).

Like in other pediatric cancer types, Mφ are an abundant immune component in Ewing sarcoma (14). Mφ are composed of multiple subtypes with different activation stages/polarization and different functions, including immunomodulation, and some specific to tissue locations (FABP4^+^ Mφ). Heterogeneity of Mφ composition and function, as described here for Ewing sarcoma, has also been reported for other pediatric and adult cancer types (44–46). We show that the immunosuppressive Mφ in Ewing sarcoma is enriched for IFN response signatures. This suggests a sustained release of proinflammatory cytokines in the Ewing sarcoma TME, which eventually leads to immune suppression and tolerance (36, 47).

Furthermore, we identify several T and NK cell subsets in the TME of Ewing sarcoma, that, like in other cancer types, can either promote or inhibit tumor progression (48–50). A subpopulation of Ewing sarcoma–associated CD8^+^ T cells shows elevated levels of dysfunction as indicated by high levels of *LAG-3* expression. CD8^+^ T-cell dysfunction has been shown in other cancers (26, 51) and may result from exhaustion, as in chronic T-cell antigen stimulation (exhaustion), or T-cell tolerance/anergy due to T-cell stimulation with low costimulatory and/or high coinhibitory signaling (52). Our data suggest that in Ewing sarcoma, prolonged exposure to tolerogenic signaling, mediated by immunosuppressive myeloid cells and/or dysfunctional DCs, is the mechanism that leads to T-cell dysfunction.

Overall, our analysis shows a higher proportion of T/NK cells relative to myeloid cells (∼1:1; Supplementary Fig. S4C) than has been previously described in IHC or transcriptome-based studies (14, 15). These inconsistencies could have been a result of either biases introduced by the CEL-Seq2 protocol (16, 17) or the freeze-thaw cycle to which the Ewing sarcoma tissue samples were subjected. However, the larger number of T and NK cells did allow us to identify and functionally characterize subtypes in Ewing sarcoma, which have not been described before. Notable, in this study, we used small needle biopsies, which cannot be easily monitored in protocols demanding a high number of viable cells, making the CEL-Seq2 protocol the approach-of-choice.

Finally, this work identifies several cell–cell interactions, utilized by Ewing sarcoma tumor cells and immune cells, to shape the Ewing sarcoma immunosuppressive microenvironment. These included interactions between Ewing sarcoma tumor cells and myeloid cells with an immunomodulatory nature, which involve dampening the antigen-presenting capacity of cDCs and polarizing Mφ to an immunosuppressive, M2-like phenotype. Several of these Ewing sarcoma tumor cell–expressed genes have been previously studied in Ewing sarcoma [*AXL, WNT5A, IGF-1R, TNFRSF10B* (TRIAL-R2/DR5)], and have been described as potential targets in Ewing sarcoma and/or to be involved in Ewing sarcoma tumor cell migration/metastatic potential (53–57).

It must be noted that our cohort includes metastatic samples, next to biopsies and resection material. Sample location, especially metastatic site versus primary location, can show differences in sample composition. The two metastatic samples included in the analysis are lymph node resections from which deliberately only the CD45^−^ fractions was single-cell sequenced (Table 1). Therefore, comparison of the microenvironment of metastatic versus primary samples was not feasible. Likewise, sample/cell number did not allow us to investigate differences in the immune microenvironment related to the observed tumor heterogeneity.

Immunotherapeutic approaches for Ewing sarcoma have shown varying successes in the preclinical setting and only few made it to clinical testing. Tumor-induced cDC dysfunction may limit the efficacy of immunotherapeutic strategies that rely on the activity of cDCs *in situ* to stimulate antitumor immunity. This could explain why several immunotherapeutic strategies for Ewing sarcoma have failed (13, 39). Furthermore, it has been previously described that patients with Ewing sarcoma fail to induce Ewing sarcoma antigen-associated immune responses (58). This could be explained by the compromised *in vivo* antigen presentation and T-cell stimulation, as well as the absence/low HLA class I expression on tumor cells. Therefore, the most feasible approach for immunotherapy to treat Ewing sarcoma would be one that is independent of HLA class I molecules and counteracts the immune suppression induced by Ewing sarcoma tumor cells. One such approach could involve chimeric antigen receptor engineering of T cells (CAR-T cells) targeting Ewing sarcoma cell-specific surface antigens, such as pregnancy-associated plasma protein-A (PAPP-A; Fig. 1D; refs. 23, 59). To ensure sustained effector function of CAR-T cells, it has to be designed with the right “build-in” T-cell stimulator, such as GMCSF/IL18 (60). Another promising treatment currently being tested in a phase II clinical trial in patients with refractory Ewing sarcoma (NCT02511132) is FANG (or Vigil) immunotherapy: autologous Ewing sarcoma tumor cells transfected with a transgene expressing recombinant human GMCSF and bifunctional short hairpin RNA against furin. This approach will create a vaccine that assists in antigen presentation and recruitment, activation, and enhanced migration of cDCs to local lymph nodes, and—most importantly—reverses immune tolerance (via furin). A phase I trial, including 12 patients with advanced/refractory Ewing sarcoma showed that the treatment was well tolerated, elicited tumor-specific systemic immune responses in all patients, and reached a 1-year OS of 75% (61).

In conclusion, this study provides a comprehensive overview of the composition and functional state of immune cells in the Ewing sarcoma TME and suggests mechanisms by which Ewing sarcoma tumor cells interact with, and shape, the immune microenvironment. In addition to enhancing the antigen-presenting capacity of cDCs, it is essential to counteract the immune suppression induced by the tumor cells.

## Supplementary Material

Extended Methods Extension of the methods related to the scRNA-seq data analysis

Supplementary Table 1 Patient and sample characteristics

Supplementary Table 2 Interactions literature references

Supplementary Table 3 Cell-cell interaction output T/NK vs. myeloid cells

Supplementary Table 4 Correlation interactions and dysfunction score CD8+ T cells

Supplementary Figure S1 Gating strategy single-cell sort into 384-well plates

Supplementary Figure S2 The single-cell architecture of Ewing sarcoma - part 1

Supplementary Figure S3 Cancer-associated fibroblast subsets in Ewing sarcoma

Supplementary Figure S4 Characterisation of Ewing sarcoma tumor cell clusters

Supplementary Figure S5 Immune cell landscape of Ewing sarcoma

Supplementary Figure S6 Lymphocytes with various degrees of dysfunction and immune-suppression

Supplementary Figure S7 Immunosuppressed and functionally impaired myeloid cells in Ewing sarcoma

Supplementary Figure S8 Comparison myeloid cells originating from Ewing sarcoma and neuroblastoma tissue samples

Supplementary Figure S9 Predicted cell-cell interactions in the Ewing sarcoma microenvironment
