# Supplementary material for: Ewing Sarcoma Single-cell Transcriptome Analysis Reveals Functionally Impaired Antigen-presenting Cells
Source: Cancer Res Commun. 2023 Oct 24;3(10):2158–69. doi: 10.1158/2767-9764.CRC-23-0027 (PMC10595530; doi:10.1158/2767-9764.CRC-23-0027)
Supplement: Extended Methods — Extension of the methods related to the scRNA-seq data analysis [file crc-23-0027-s01.pdf]

EXTENDED METHODS

Mapping and filtering

The Sharq pipeline was used to process the sequencing data as previously described (1). Mapping was performed using STAR (version 2.6.1), on the Genome Reference Consortium GRCh38. Read assignment was performed with featureCounts (version 1.5.2), using a gene annotation based on GENCODE version 26. External RNA controls (ERCCS) and transcripts mapping to the mitochondrial genome were removed from all cells. The same for transcripts of genes identified based on high correlation with the hemoglobin genes expression, as previously described by Hanemaaijer et al.(2) To avoid contamination, wells where the inserts were mapping over 15-fold to non-exonic as compared to exonic regions, cells with mitochondrial-encoded transcripts over 80% of nuclear transcripts, cells expressing over 5% of their total transcriptome erythroid marker genes were removed. In addition, cells with less than 800 transcripts, or more than $150^\wedge10^3$ transcripts, were removed from the dataset. Genes with low expression, that is either having less than 5 cells expressing the gene or less than two cells with less than two transcripts, were removed. To improve cross-sample comparisons, ambient mRNA contamination in individual cells was estimated and removed using DecontX (3). DecontX was run for all samples (batches) individually. Removal of cells with less than 800 transcripts was repeated on the decontaminated counts matrix.

scRNA-seq quality control and data processing

Unique transcript counts were normalized using sctransform (4) and analyzed using the Seurat R package (version 4.0.0) (5). From the top 3000 variable genes, genes associated with cell cycle phase, dissociation stress (heat shock and chaperone proteins according to GO:0006986), sex (XIST, TSIX, and Y chromosome-specific genes), and activity (ribosomal protein genes according to GO:0022626) were removed from the list of variable genes to avoid biases in cell clustering, as described before (6).

Principle component analyses were performed using the filtered variable gene lists. To study the main cell types in our dataset, the first 30 principal components (PCs) were used to calculate dimensionality reduction using UMAP and to perform clustering with a resolution of 1, using the Louvain algorithm. For CAF- and immune cell-focused analysis, the respective clusters were subsetted. In-depth analysis of the CAFs subsequently involved 20 PCs for UMAP dimensional reduction and clustering, using a resolution of 0.1. In-depth analysis of the myeloid cells involved 30 PCs and k.param set to 15 for UMAP dimensional reduction and clustering, using a resolution of 0.8. Lastly, in-depth analysis of the T/NK cells involved renormalization of the data using SCTransfrom, followed by 25 PCs and k.param set to 15 for UMAP dimensional reduction and clustering, using a resolution of 1.6.

Differential gene expression analysis

Cluster-specific genes were identified using *FindAllMarkers* from the Seurat package (5), using the Wilcoxon test with 1.5-fold change expression cutoff and 5% Bonferroni multiple testing corrected statistical significance cutoff. Only positive markers were considered. Volcano plots were created with the R package EnhancedVolcano.

Cell annotation using external data

Cluster annotations were guided by SingeR (versions 1.2.4) (7), using the *HumanPrimaryCellAtlas* reference dataset (8) to annotate the main cell types, and using the *MonacoImmuneData* reference dataset (9) to annotate the T/NK cell subset. Cell annotations were further refined by consulting the cluster specific upregulated differentially expressed genes and known cell type-specific marker genes from previous studies (Table M1). Malignant cells were identified using three criteria: (1) their inferred copy number profiles (inferCNV; see below); (2) under-expression or absence of different non-malignant cell type markers; and (3) high expression of EwS-associated genes, based on Hu-Lieskovan, et al. (10) (Supplementary Fig S3a).

Table M1. Overview of marker genes used in annotating cell subsets

Cell subset	Marker genes
Main	
Immune cells	*PTPRC*
Mesenchymal-like cells	*COL1A1, COL3A1*
Endothelial cells	*PECAM1, VWF*
Tumor cells	*CD99, CAV1, CCND1, HES1, KDSR, PAPPA*
Detailed immune	
(plasma) B cells	*MS4A1, CD19, CD79A, GZMB, LILRA4*
T cells	*CD3E, CD4, CD8A, GZMB, FOXP3, TCF7, SELL, LEF1, TRDC, TRGC1, TRGC2*
NK cells	*GNLY, PRF1, NKG7*
Macrophages	*CD68, CD74, CD163, LYZ, MRC1, MSR1, S100A8/9, FCN1, CD14, FCGR3A*
Dendritic cells	*CD1C, FCER1A, LAMP3, CLEC9A, CLEC10A*
Mast cells	*CPA3, TPSAB1*

Refs: (11–17)

Cellular composition analysis

For assessing the composition of cell types of the individual tumor samples, we solely included unbiasedly sorted plates (DAPI & DRAQ5, annotated as “live” in Supplementary Table 1) in case of full sample composition or unbiasedly sorted and CD45+ enriched plates (“CD45+” in Supplementary Table 1) in case of immune composition. For immune cell composition analysis, samples with <=20 immune cells were excluded. Bar plots were generated using ggplot2.

Copy Number Variation analysis

We identified malignant cells by inferring the somatic large-scale chromosomal copy number alterations using inferCNV version 1.6.0. We used the endothelial, mesenchyme and immune cells as a healthy reference. The gene ordering file, which contains the chromosomal start and end positions for each gene, was created from ENSEMBL using the biomaRt R package (version 2.46.3) (18). To run inferCNV, we used the following parameters: a cutoff of 0.1, clustered cells into the annotated clusters, denoised the output and applied an i3 hidden Markov model.

For the phylogenetic analysis, InferCNV scores were averaged across chromosome arms and then scaled and centered (i.e., z-transformed) based on the healthy cell clusters. Z-scores over 2.5 were assigned as gains (red), while below -2.5 as losses (blue).

Gene signatures

For the identification of the CAF subsets and immune cell phenotypes, module scores were calculated using the AddModuleScore function of Seurat using the following gene signatures. CAF subsets were identified using the top 30 differentially expressed genes of the 6 distinct CAF subsets described by Luo, et al. (19), as well as the top 30 differentially expressed genes between CAFS and normal fibroblasts. Macrophage phenotypes were determined using the M1 and M2 gene signatures from Cheng, et al. (20) and pro- and anti-inflammatory gene signatures from Wauters, et al. (21). For the analysis of T and NK cell function, the dysfunction gene signature from Jerby-Arnon, et al. (22). and Tirosh, et al. (23) was used. For comparing cDC cell state between neuroblastoma and EwS, the curated MSigDB gene set “LINDSTEDT_DENDRITIC_CELL_MATURATION_B” was used (24). This gene set includes up-regulated genes both at 8 hours and 48 hours in response to inflammatory stimuli.

Gene set enrichment analysis

Differential expression analysis of the T/NK subsets was performed with FindAllMarkers using adjusted parameters: logfc.threshold=0, min.pct = 0, min.cells.feature = 0, min.cells.group = 0. Genes were pre-ranked by their Fold Change and GSEA was performed using the fgsea package. Gene sets were obtained from MSigDB version 7.2.

For identification of EwS-associated T cell subsets, the logFC-ranked gene lists of the T/NK subset were used for GSEA, where gene signatures of blood-/normal tissue/ tumor-associates T cells subsets from Zheng, et al. were used as gene sets (25).

Cell-cell interaction analysis

The CellChat algoritm was applied to unbiasedly model ligand-receptor interactions, using the curated ligand-receptor database of CellPhoneDB (www.cellphonedb.org) (26). To identify myeloid–CD8$^+$ T cell interactions that were positively correlating with the dysfunction score of CD8$^+$ T cells, we selected the genes 1) that were involved in a significant interaction ($p<0.05$) between myeloid cell subsets and CD8$^+$ T cells and were expressed by the respective myeloid cell subset, and 2) that positively correlated (>0.5) with the dysfunction score of CD8$^+$ T cells in the same sample.

References

1. Candelli T, Lijnzaad P, Muraro MJ, Kerstens H, Oudenaarden A Van, Margaritis T, et al. Sharq A versatile preprocessing and QC pipeline for Single Cell RNA-seq.
2. Hanemaaijer ES, Margaritis T, Sanders K, Bos FL, Candelli T, Al-Saati H, et al. Single-cell atlas of developing murine adrenal gland reveals relation of Schwann cell precursor signature to neuroblastoma phenotype. Proc Natl Acad Sci. 2021;118:e2022350118.
3. Yang S, Corbett SE, Koga Y, Wang Z, Johnson WE, Yajima M, et al. Decontamination of ambient RNA in single-cell RNA-seq with DecontX. Genome Biol. Genome Biology; 2020;21:57.
4. Hafemeister C, Satija R. Normalization and variance stabilization of single-cell RNA-seq data using regularized negative binomial regression. Genome Biol. 2019;20:296.
5. Butler A, Hoffman P, Smibert P, Papalexi E, Satija R. Integrating single-cell transcriptomic data across different conditions, technologies, and species. Nat Biotechnol. 2018;36:411–20.
6. Calandrini C, Schutgens F, Oka R, Margaritis T, Candelli T, Mathijsen L, et al. An organoid biobank for childhood kidney cancers that captures disease and tissue heterogeneity. Nat Commun. Springer US; 2020;11.
7. Aran D, Looney AP, Liu L, Wu E, Fong V, Hsu A, et al. Reference-based analysis of lung single-cell sequencing reveals a transitional profibrotic macrophage. Nat Immunol. Springer US; 2019;20:163–72.
8. Mabbott NA, Baillie JK, Brown H, Freeman TC, Hume DA. An expression atlas of human primary cells: Inference of gene function from coexpression networks. BMC Genomics. 2013;14.
9. Monaco G, Lee B, Xu W, Mustafah S, Hwang YY, Carré C, et al. RNA-Seq Signatures Normalized by mRNA Abundance Allow Absolute Deconvolution of Human Immune Cell Types. Cell Rep. 2019;26:1627-1640.e7.
10. Hu-Lieskovan S, Zhang J, Wu L, Shimada H, Schofield DE, Triche TJ. EWS-FLI1 fusion protein up-regulates critical genes in neural crest development and is responsible for the observed phenotype of Ewing's family of tumors. Cancer Res. 2005;65:4633–44.
11. Heitzeneder S, Sotillo E, Shern JF, Sindiri S, Xu P, Jones R, et al. Pregnancy-Associated Plasma Protein-A (PAPP-A) in Ewing Sarcoma: Role in Tumor Growth and Immune Evasion. J Natl Cancer Inst. 2019;111:970–82.
12. Li H, van der Leun AM, Yofe I, Lubling Y, Gelbard-Solodkin D, van Akkooi ACJ, et al. Dysfunctional CD8 T Cells Form a Proliferative, Dynamically Regulated Compartment within Human Melanoma. Cell. Elsevier Inc.; 2019;176:775-789.e18.
13. Town J, Pais H, Harrison S, Stead LF, Bataille C, Bunjobpol W, et al. Exploring the surfaceome of Ewing sarcoma identifies a new and unique therapeutic target. 2016;1.
14. Zavidij O, Haradhvala NJ, Mouhieddine TH, Sklavenitis-Pistofidis R, Cai S, Reidy M, et al. Single-cell RNA sequencing reveals compromised immune microenvironment in precursor stages of multiple myeloma. Nat Cancer. 2020;1:493–506.

15. Szabo PA, Levitin HM, Miron M, Snyder ME, Senda T, Yuan J, et al. Single-cell transcriptomics of human T cells reveals tissue and activation signatures in health and disease. Nat Commun. Springer US; 2019;10:4706.

16. Zhao J, Guo C, Xiong F, Yu J, Ge J, Wang H, et al. Single cell RNA-seq reveals the landscape of tumor and infiltrating immune cells in nasopharyngeal cancer. Cancer Lett. Elsevier; 2020;477:131–43.

17. Aynaud MM, Mirabeau O, Gruel N, Grossetête S, Boeva V, Durand S, et al. Transcriptional Programs Define Intratumoral Heterogeneity of Ewing Sarcoma at Single-Cell Resolution. Cell Rep. 2020;30:1767-1779.e6.

18. Durinck S, Spellman PT, Birney E, Huber W. Mapping identifiers for the integration of genomic datasets with the R/Bioconductor package biomaRt. Nat Protoc. 2009;4:1184–91.

19. Luo H, Xia X, Huang L Bin, An H, Cao M, Kim GD, et al. Pan-cancer single-cell analysis reveals the heterogeneity and plasticity of cancer-associated fibroblasts in the tumor microenvironment. Nat Commun. 2022;13.

20. Cheng S, Li Z, Gao R, Xing B, Gao Y, Yang Y, et al. A pan-cancer single-cell transcriptional atlas of tumor infiltrating myeloid cells. Cell. Elsevier Inc.; 2021;184:792-809.e23.

21. Wauters E, Van Mol P, Garg AD, Jansen S, Van Herck Y, Vanderbeke L, et al. Discriminating mild from critical COVID-19 by innate and adaptive immune single-cell profiling of bronchoalveolar lavages. Cell Res. 2021;31:272–90.

22. Jerby-Arnon L, Shah P, Cuoco MS, Rodman C, Su MJ, Melms JC, et al. A Cancer Cell Program Promotes T Cell Exclusion and Resistance to Checkpoint Blockade. Cell. 2018;175:984-997.e24.

23. Tirosh I, Izar B, Prakadan SM, Wadsworth MH, Treacy D, Trombetta JJ, et al. Dissecting the multicellular ecosystem of metastatic melanoma by single-cell RNA-seq. Science. 2016;352:189–96.

24. Lindstedt M, Johansson-Lindbom B, Borrebaeck CAK. Global reprogramming of dendritic cells in response to a concerted action of inflammatory mediators. Int Immunol. 2002;14:1203–13.

25. Zheng L, Qin S, Si W, Wang A, Xing B, Gao R, et al. Pan-cancer single-cell landscape of tumor-infiltrating T cells. Science. 2021;374:abe6474.

26. Jin S, Guerrero-Juarez CF, Zhang L, Chang I, Ramos R, Kuan C-H, et al. Inference and analysis of cell-cell communication using CellChat. Nat Commun. 2021;12:1088.
