# Supplementary material for: Ewing Sarcoma Single-cell Transcriptome Analysis Reveals Functionally Impaired Antigen-presenting Cells
Source: Cancer Res Commun. 2023 Oct 24;3(10):2158–69. doi: 10.1158/2767-9764.CRC-23-0027 (PMC10595530; doi:10.1158/2767-9764.CRC-23-0027)
Supplement: Supplementary Figure S1 — Gating strategy single-cell sort into 384-well plates [file crc-23-0027-s06.pdf]

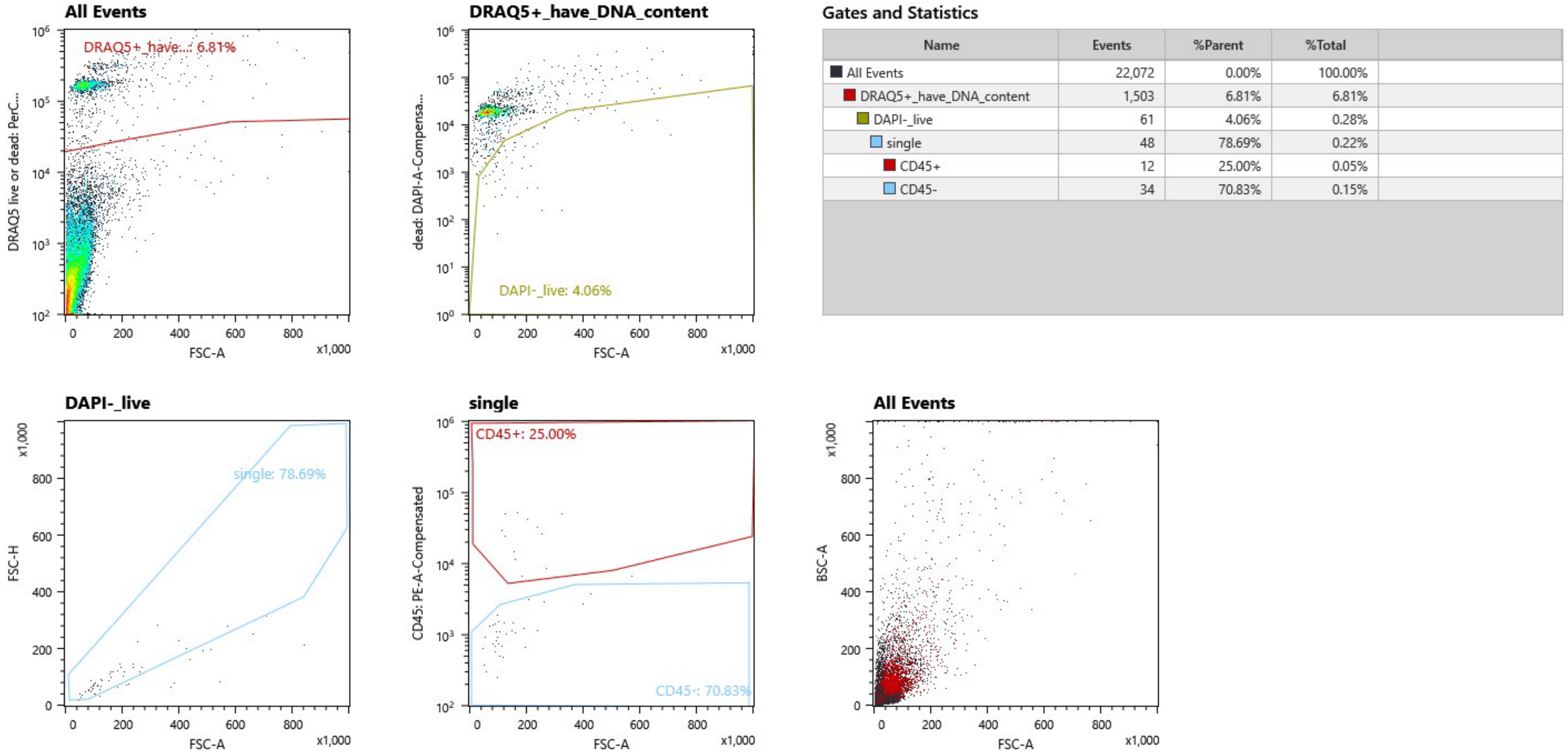

Name	Events	%Parent	%Total
All Events	22,072	0.00%	100.00%
DRAQ5+_have_DNA_content	1,503	6.81%	6.81%
DAPI-_live	61	4.06%	0.28%
single	48	78.69%	0.22%
CD45+	12	25.00%	0.05%
CD45-	34	70.83%	0.15%

Figure S1. Gating strategy single-cell sort into 384-well plates

Viable single cells were sorted based on forward/side-scatter properties, DAPI-, DRAQ5-, and CD45 staining, using FACS.
