# Supplementary figures and images for: Ewing Sarcoma Single-cell Transcriptome Analysis Reveals Functionally Impaired Antigen-presenting Cells
Source: Cancer Res Commun. 2023 Oct 24;3(10):2158–69. doi: 10.1158/2767-9764.CRC-23-0027 (PMC10595530; doi:10.1158/2767-9764.CRC-23-0027)

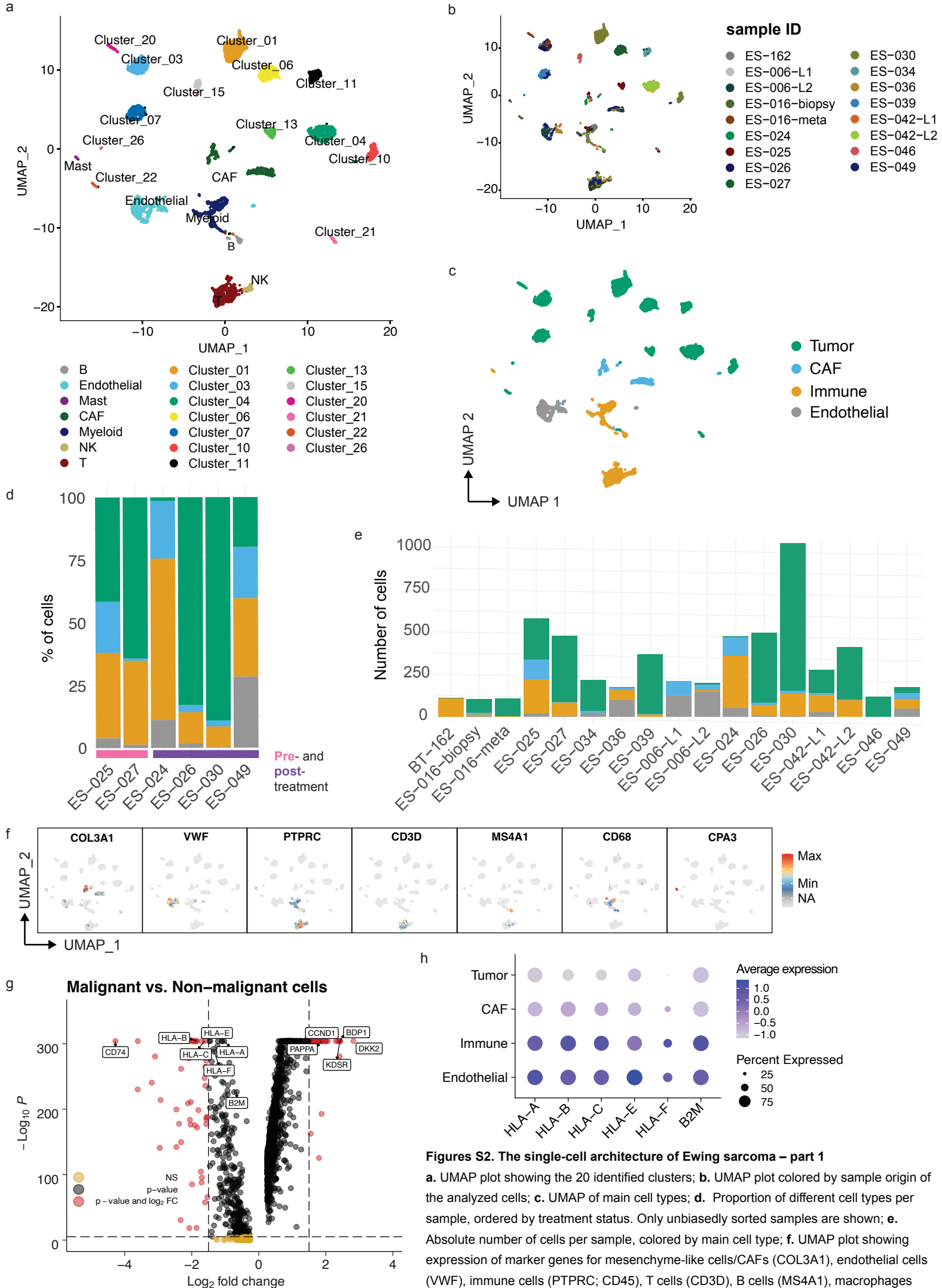

Supplement: Supplementary Figure S2 — The single-cell architecture of Ewing sarcoma - part 1 [file crc-23-0027-s07.pdf]

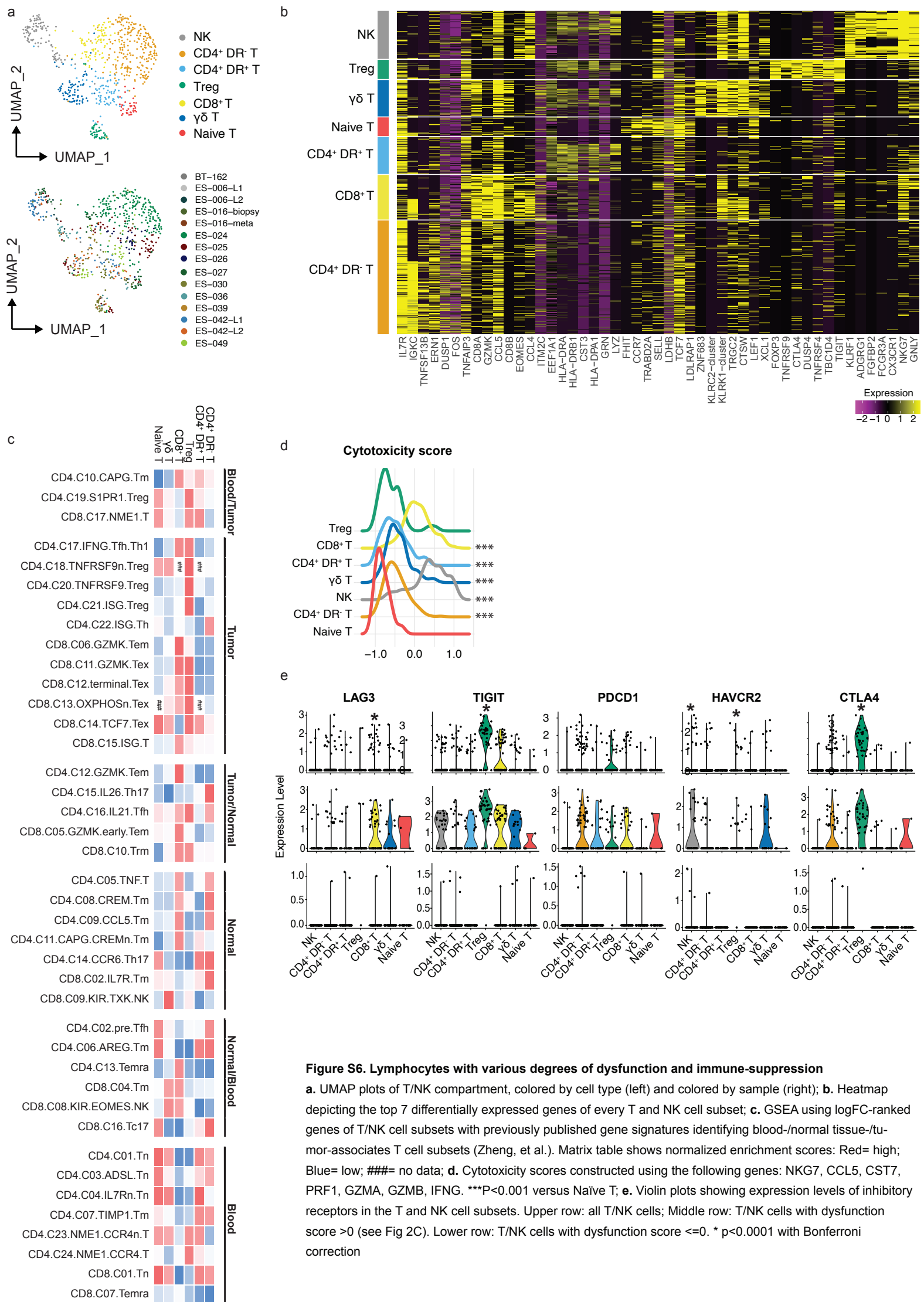

Supplement: Supplementary Figure S6 — Lymphocytes with various degrees of dysfunction and immune-suppression [file crc-23-0027-s11.pdf]

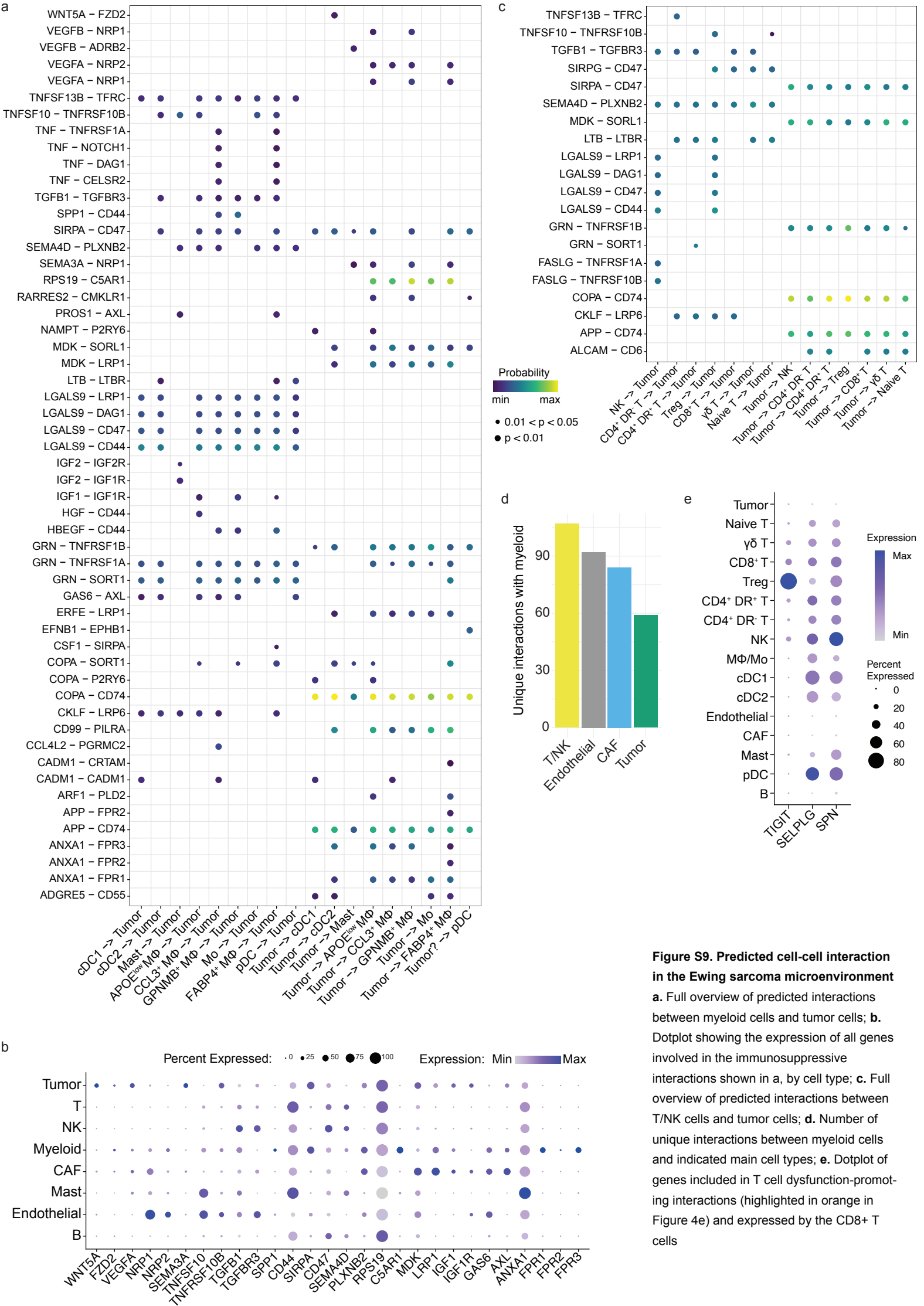

Supplement: Supplementary Figure S9 — Predicted cell-cell interactions in the Ewing sarcoma microenvironment [file crc-23-0027-s14.pdf]
