# Supplementary material for: Ewing Sarcoma Single-cell Transcriptome Analysis Reveals Functionally Impaired Antigen-presenting Cells
Source: Cancer Res Commun. 2023 Oct 24;3(10):2158–69. doi: 10.1158/2767-9764.CRC-23-0027 (PMC10595530; doi:10.1158/2767-9764.CRC-23-0027)
Supplement: Supplementary Figure S3 — Cancer-associated fibroblast subsets in Ewing sarcoma [file crc-23-0027-s08.pdf]

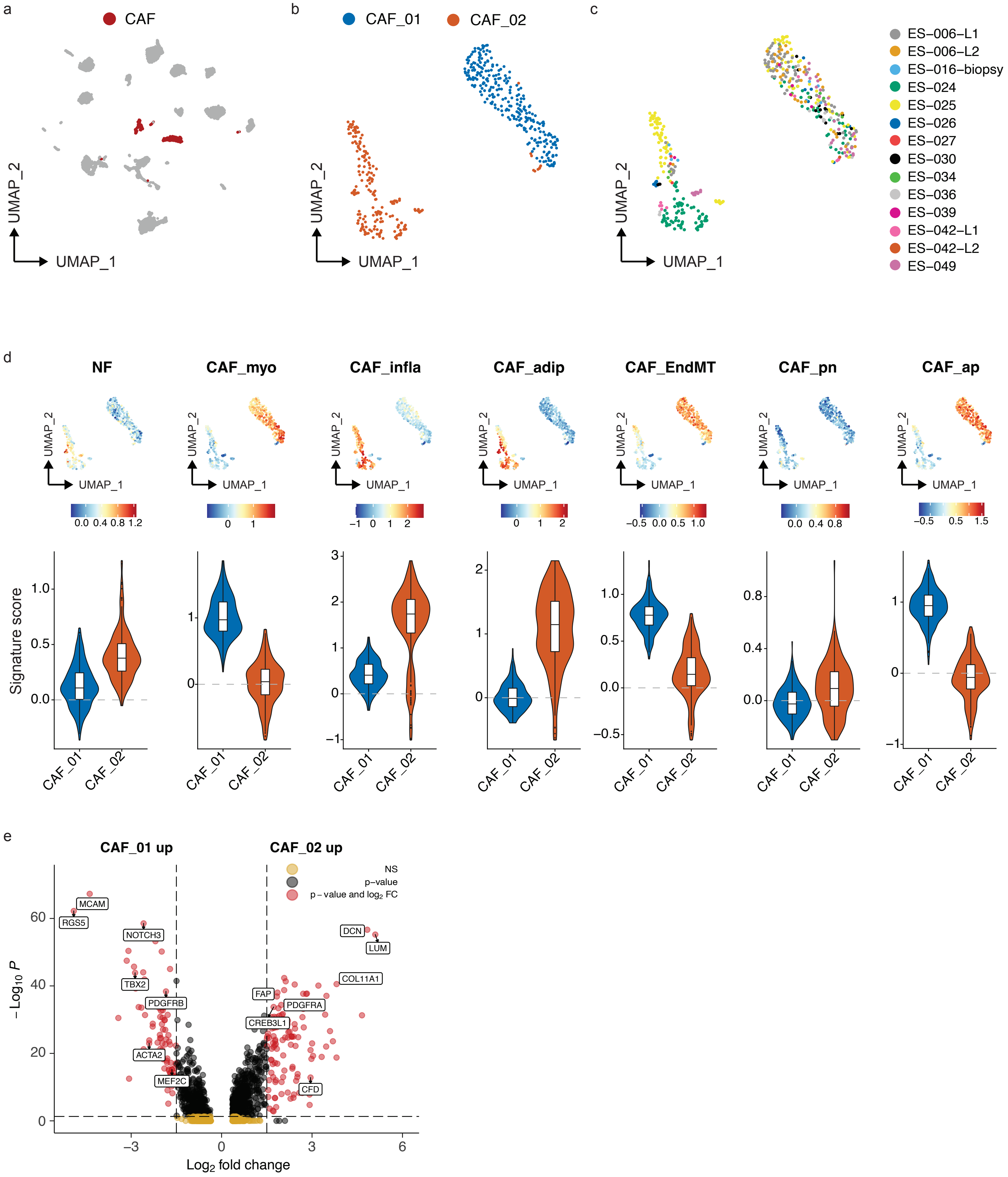

Fig S3. Cancer-associated fibroblast subsets in Ewing sarcoma

a. UMAP plot of the full dataset where cancer-associate fibroblasts (CAF) are highlighted in red; **b.** UMAP plot of subsetted CAFs colored by subset; c. UMAP plot of CAFs colored by sample; **d.** Feature plots (upper part) and violin plots (lower part) showing CAF and normal fibroblast signature scores; **e.** Volcano plot of differentially expressed genes (padj <0.05) between the two CAF subsets. Signatures from Luo, et al. (2022) Nat Comm. NF: normal fibroblasts; CAF_myo: cancer-associated myofibroblasts; CAF_infla: inflammatory CAFs; CAF_adip: adipogenic CAFs; CAF_EndMT: endothelial-to-mesenchymal transition CAFs; CAF_pn: peripheral nerve-like CAFs; CAF_ap: antigen presenting CAFs
