# Supplementary material for: Ewing Sarcoma Single-cell Transcriptome Analysis Reveals Functionally Impaired Antigen-presenting Cells
Source: Cancer Res Commun. 2023 Oct 24;3(10):2158–69. doi: 10.1158/2767-9764.CRC-23-0027 (PMC10595530; doi:10.1158/2767-9764.CRC-23-0027)
Supplement: Supplementary Figure S4 — Characterisation of Ewing sarcoma tumor cell clusters [file crc-23-0027-s09.pdf]

a

b

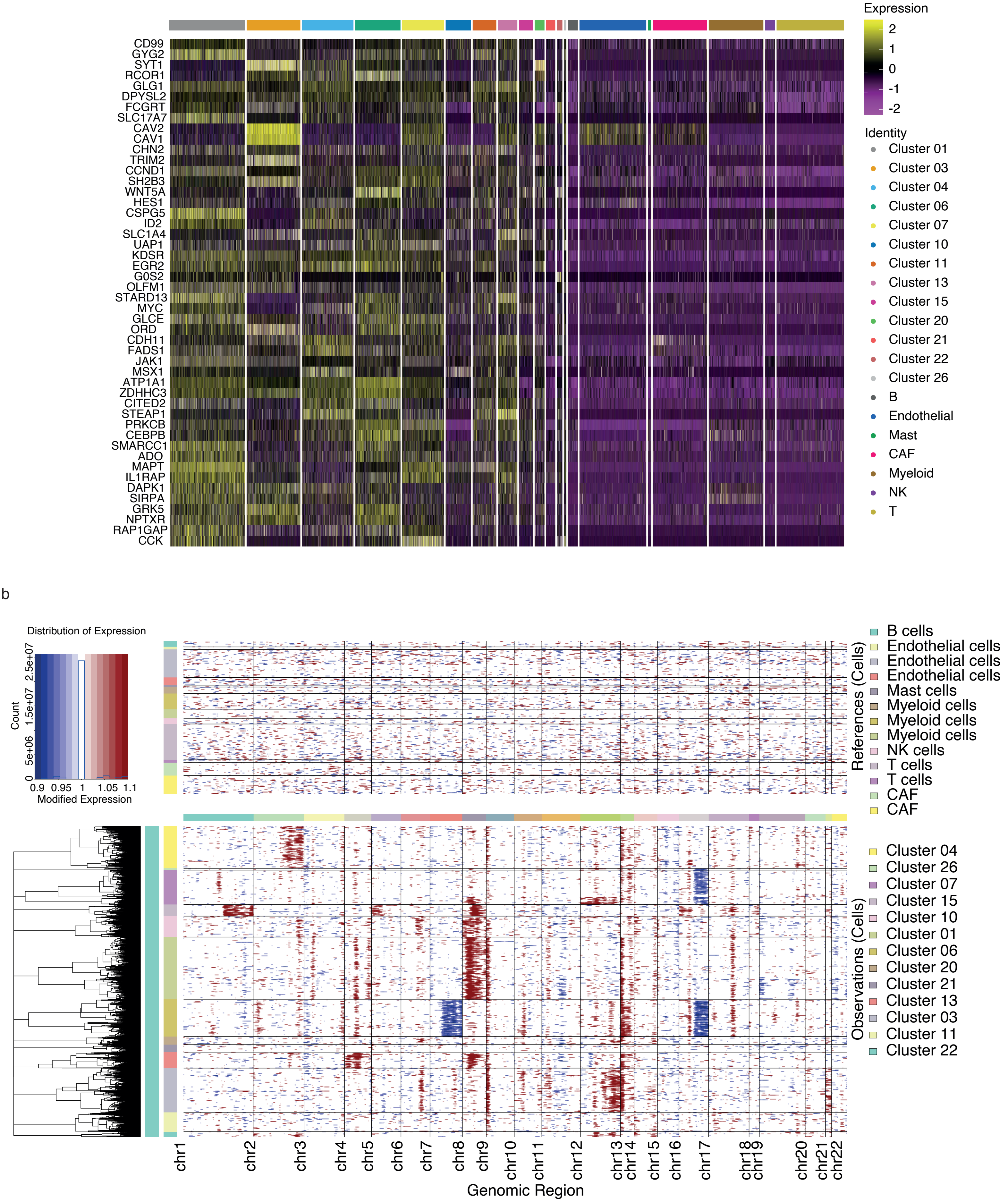

Figure S4. Characterization of Ewing sarcoma tumor cell clusters

a. Heatmap depicting expression of Ewing-associated genes, based on Hu-Lieskovan et al. in tumor- and healthy cell populations; **b.** Heatmap depicting CNV profiles of each cell inferred from inferCNV analysis; chromosomes are lined up on the horizontal axis and cell clusters on the vertical axis. Immune cells, endothelial cells, and cancer-associated fibroblasts on the upper panel were used as reference healthy cell clusters
