# Supplementary material for: Ewing Sarcoma Single-cell Transcriptome Analysis Reveals Functionally Impaired Antigen-presenting Cells
Source: Cancer Res Commun. 2023 Oct 24;3(10):2158–69. doi: 10.1158/2767-9764.CRC-23-0027 (PMC10595530; doi:10.1158/2767-9764.CRC-23-0027)
Supplement: Supplementary Figure S5 — Immune cell landscape of Ewing sarcoma [file crc-23-0027-s10.pdf]

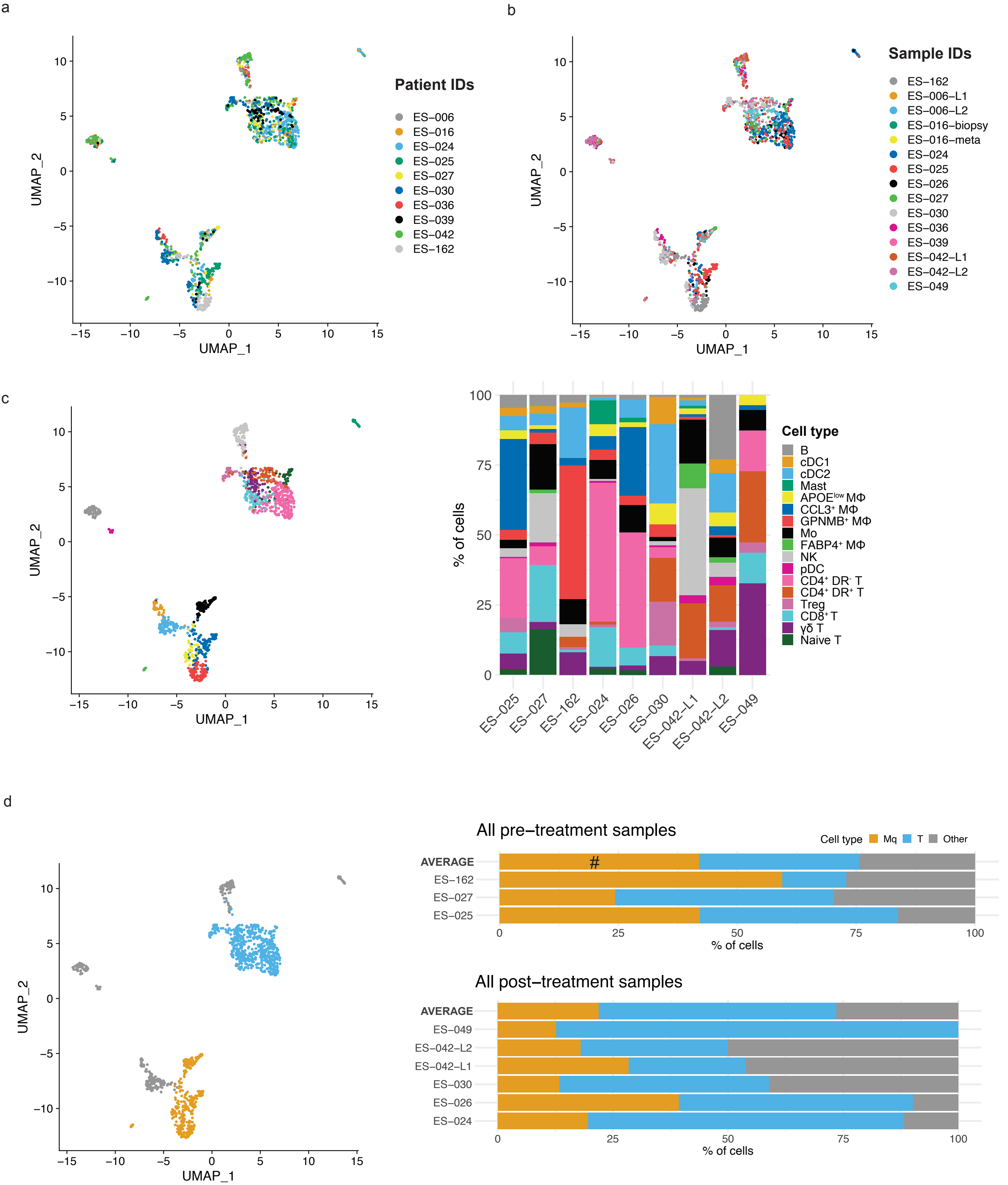

Figure S5. Immune cell landscape of Ewing sarcoma

a. UMAP plot of the immune cell compartment where cells are colored by patient; **b.** UMAP plot immune cell compartment with cells colored by sample; **c.** UMAP and barplot showing the composition of the identified immune cell subsets; **d.** UMAP and barplot showing the composition of the identified immune cells subsets, categorized by macrophages (Mq), T cells and other cells. # trend (p=0.1) between pre- and post-treatment samples
