# Supplementary material for: Ewing Sarcoma Single-cell Transcriptome Analysis Reveals Functionally Impaired Antigen-presenting Cells
Source: Cancer Res Commun. 2023 Oct 24;3(10):2158–69. doi: 10.1158/2767-9764.CRC-23-0027 (PMC10595530; doi:10.1158/2767-9764.CRC-23-0027)
Supplement: Supplementary Figure S7 — Immunosuppressed and functionally impaired myeloid cells in Ewing sarcoma [file crc-23-0027-s12.pdf]

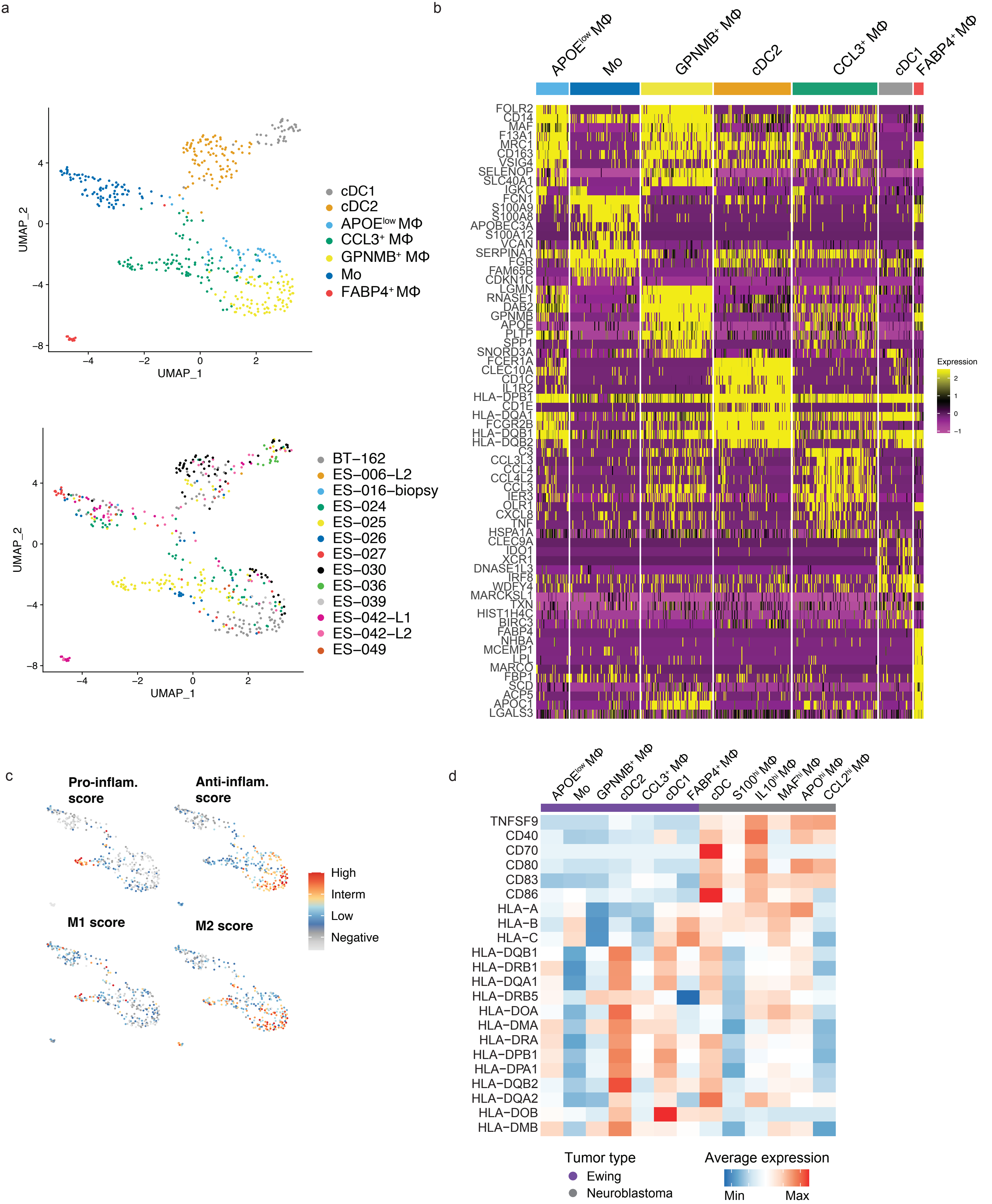

Figure S7. Immunosuppressed and functionally impaired myeloid cells in Ewing sarcoma

a. UMAP plots of myeloid compartment, colored by cell type (upper) and colored by sample (lower); **b.** Heatmap depicting the top 10 differentially expressed genes of every myeloid cell subset; **c.** UMAP plots of the subsetted macrophage populations, colored by pro- and anti-inflammatory signature scores, and colored by M1 and M2 signature scores; **d.** Heatmap showing average expression of genes involved in co-stimulatory and antigen presentation capacity of conventional dendritic cells and macrophages, involving the myeloid cell populations of the Ewing and neuroblastoma dataset. The neuroblastoma cDC subset includes both cDC1 and cDC2 cells
