# Supplementary material for: Ewing Sarcoma Single-cell Transcriptome Analysis Reveals Functionally Impaired Antigen-presenting Cells
Source: Cancer Res Commun. 2023 Oct 24;3(10):2158–69. doi: 10.1158/2767-9764.CRC-23-0027 (PMC10595530; doi:10.1158/2767-9764.CRC-23-0027)
Supplement: Supplementary Figure S8 — Comparison myeloid cells originating from Ewing sarcoma and neuroblastoma tissue samples [file crc-23-0027-s13.pdf]

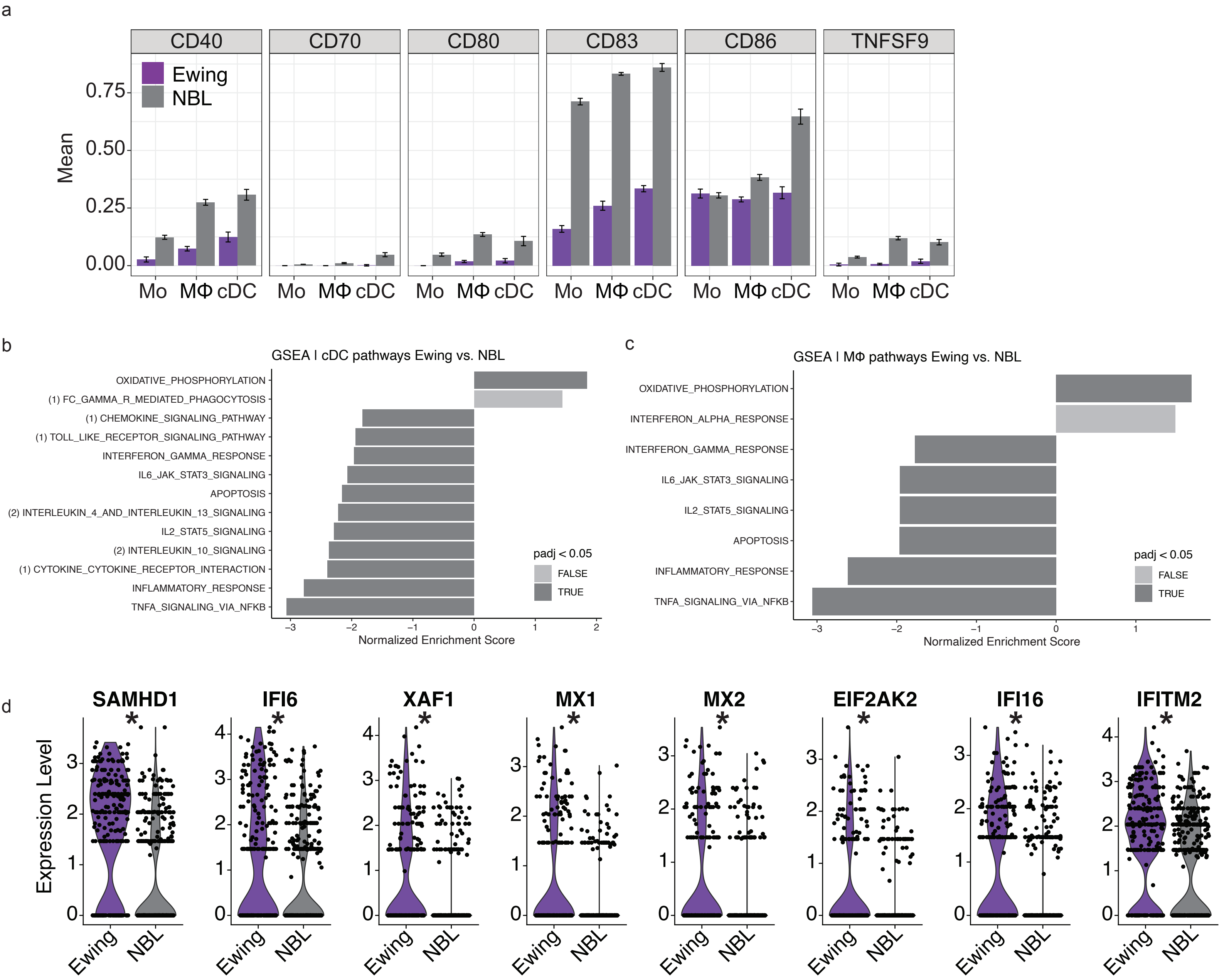

Figure S8. Comparing myeloid cells originating from Ewing sarcoma and neuroblastoma tissue samples

a. Barplot showing percentage of co-stimulatory gene-expressing Mo, Mφ and cDCs. Neuroblastoma (NBL; Kildisiute, et al.) data included as comparative group. Mean percentages and error bars are plotted for 10 iterations after downsampling the data to 3000 transcript/cell to account for differences in sequencing depth; **b.** GSEA of cDC in Ewing sarcoma compared to neuroblastoma tissue samples. (1) indicates KEGG genesets, (2) indicates REAC-TOME gene sets and remainder are HALLMARK genesets; **c.** GSEA of differentiated macrophages in Ewing sarcoma compared to neuroblastoma tissue samples. HALLMARK genesets are shown; **d.** Violin plot showing expression levels of interferon responsive genes in the macrophages. * $p<0.0001$ with Bonferroni correction
